# A 220 GHz Integrated Sensing and Communication System Based on Orthogonal-Polarization Multiplexing

**DOI:** 10.3390/mi17101109

**Published:** 2026-09-22

**Authors:** Jicong Zhang, Zhankun Tang, Shuhang Shi, Ke Liu, Yinian Feng, Hui Peng, Bo Zhang

**Affiliations:** 1 School of Electronic Science and Engineering, University of Electronic Science and Technology of China, Chengdu 611731, China; 2 Nation Key Laboratory of Science and Technology on Communications, University of Electronic Science and Technology of China, Chengdu 611731, China; 3 College of Electronic and Communication Engineering, Kashi University, Kashi 844000, China; 4 Shenzhen Institute for Advanced Study, University of Electronic Science and Technology of China, Shenzhen 518038, China

**Keywords:** terahertz communications, orthogonal-polarization multiplexing, integrated sensing and communication, orthomode transducer, radar ranging

## Abstract

A 220 GHz integrated sensing and communication (ISAC) system based on orthogonal-polarization multiplexing is presented for simultaneous high-speed wireless communication and radar ranging. To achieve broadband operation, low insertion loss, and high polarization isolation, a dual-symmetric reverse-coupled orthomode transducer (OMT) is designed and fabricated. The OMT covers 170–260 GHz, with a measured return loss better than 15 dB and polarization isolation exceeding 45 dB, while relaxing the required fabrication tolerance to 10 μm. The OMT is integrated with solid-state frequency-conversion circuits, amplifiers, waveguide components, and a dual-polarized antenna to construct a dual-polarization transmit front end. The resulting system achieves polarization isolation exceeding 25 dB between the communication and radar channels over 204–222 GHz, together with a receiver noise figure of 9.8 dB at 220 GHz. Using coherent-reference techniques and real-time baseband processing, the system achieves a data rate of 2 Gbit/s over a distance of 0.25 m, with a bit error rate below $5\times 10^{-8}$. Radar ranging experiments using a single corner-reflector target at distances of 0.25 and 0.40 m yielded a maximum observed absolute ranging error of 1 cm. These results demonstrate the feasibility of orthogonal-polarization multiplexing for jointly supporting high-speed THz communication and centimeter-level radar sensing.

## 1. Introduction

The terahertz (THz) band, generally defined as the frequency range from 0.1 to 10 THz, lies between the microwave and infrared regions of the electromagnetic spectrum. Owing to its short wavelength and abundant spectral resources, THz waves offer significant potential for ultra-high-speed and ultra-broadband wireless communications. Compared with conventional microwave and millimeter-wave systems, THz communication provides substantially wider bandwidths and can support data rates of tens or even hundreds of gigabits per second. Therefore, THz wireless communication is considered one of the most promising technologies for future sixth-generation (6G) communication systems [1,2,3,4,5].

Solid-state technology is an important approach for implementing THz communication systems because it offers high reliability, flexible frequency-conversion capability, and room-temperature operation, while allowing relatively compact circuit implementations [6,7,8,9]. In recent years, discrete THz front-end systems based on waveguide components and Schottky-diode circuits have been widely investigated. Various key functional components, including mixers [10,11], amplifiers [12], frequency multipliers [13,14], filters [15], and duplexers, have been developed and incorporated into high-speed wireless communication systems. Compared with highly integrated chip-based front ends, discrete solid-state front ends can provide greater flexibility in system configuration and may offer higher output power or lower conversion loss in certain frequency ranges. In 2017, Northrop Grumman Corporation reported a 666 GHz all-electronic communication system. Using QPSK modulation, the system attained a data rate of 9.5 Gbit/s at a wireless transmission distance of 590 m [16]. These results demonstrate the feasibility of using discrete THz front ends for high-capacity wireless transmission over practical distances. However, many existing systems either employ single-polarization transmission or use frequency-domain multiplexing without fully exploiting the polarization domain. Consequently, their capacity and spectral efficiency remain constrained by the available bandwidth, the number of multiplexed channels, and the performance of each individual channel.

Orthogonal-polarization multiplexing provides an effective means of overcoming these bandwidth and spectral-efficiency limitations. This technique enables two independent data streams to be transmitted through the same spatial channel using mutually orthogonal polarization modes. Compared with single-polarization transmission, it can improve spectral efficiency and increase system capacity without a proportional increase in occupied bandwidth. Several experimental demonstrations have verified the potential of polarization multiplexing for high-capacity THz communication. A 300 GHz dual-polarization link achieved a total data rate of 102.4 Gbit/s over a distance of 500 m [17]. Another 300 GHz orthogonal-polarization-multiplexed system achieved a data rate exceeding 100 Gbit/s with a polarization isolation better than 25 dB [18]. Nevertheless, compared with conventional single-polarization systems, polarization-multiplexed systems impose more stringent requirements on the isolation between the two orthogonal channels. In an ISAC system, insufficient isolation may cause leakage between the communication and radar channels, thereby degrading both communication and sensing performance.

The orthomode transducer (OMT) is a key component of an orthogonal-polarization communication front end because it separates and combines two orthogonally polarized signals [19]. The isolation of the OMT directly affects polarization crosstalk and, consequently, the signal-to-interference-plus-noise ratio, error vector magnitude, bit error rate, and overall communication quality. Based on their geometrical symmetry, OMTs can generally be classified into doubly symmetric, singly symmetric, and asymmetric structures [20,21,22]. Doubly symmetric OMTs, such as turnstile-type structures, generally provide broad bandwidth and high isolation; however, their complicated configurations impose stringent requirements on THz fabrication and assembly. Singly symmetric OMTs, such as Boifot-type structures, offer a tradeoff among bandwidth, isolation, and structural complexity. Asymmetric OMTs are easier to fabricate and are more suitable for high-frequency applications, but they generally provide poorer suppression of higher-order modes and lower polarization isolation. Therefore, simultaneously achieving a compact structure, broad bandwidth, low insertion loss, and high isolation remains challenging for THz OMT design.

In this paper, a 220 GHz integrated sensing and communication (ISAC) system based on orthogonal-polarization multiplexing is presented. A high-isolation OMT is designed to separate and combine two orthogonally polarized signals while suppressing polarization crosstalk. The proposed transceiver front end integrates solid-state frequency-conversion circuits, amplifiers, waveguide components, and polarization-separation structures. The circuit architecture and key components are analyzed and optimized, and a complete wireless ISAC link is established for experimental verification. The measured results demonstrate a polarization isolation exceeding 25 dB between the communication and radar channels, a communication data rate of 2 Gbit/s over a distance of 0.25 m, and centimeter-level radar ranging accuracy. The demonstrated architecture illustrates the potential of orthogonal-polarization multiplexing for improving the capacity and spectral efficiency of discrete THz front-end systems and provides an experimental basis for future THz ISAC applications.

## 2. System Architecture and Performance Specifications of the 220 GHz Integrated Sensing and Communication System

The overall architecture of the proposed 220 GHz integrated sensing and communication (ISAC) system is shown in Figure 1. The system consists primarily of a real-time communication baseband-processing platform, an intermediate-frequency (IF) conversion module, a radar-ranging baseband-processing platform, a dual-polarization THz transmit front end, and two single-polarization THz receive front ends. The system employs an orthogonal-polarization multiplexing architecture in which the communication and radar signals share the same frequency band but are assigned to two orthogonal polarization states. Consequently, the two signals are separated by polarization-selective antennas and the orthomode transducer (OMT), and no bandpass filters are required for channel separation.

At the transmitter, the real-time communication baseband processing platform generates the modulated communication baseband signal (1.6±0.5 GHz). This signal is then upconverted by the intermediate-frequency up-conversion module to generate the communication intermediate-frequency (IF) signal (5.4±0.5 GHz), which is fed into the communication channel of the orthogonal-polarization multiplexed transmit front end. There, it is upconverted with the communication local-oscillator (LO) carrier signal to generate a vertically polarized terahertz communication signal (220±0.5 GHz). Meanwhile, the radar ranging baseband processing platform directly generates the modulated radar IF signal (5.4±0.85 GHz). This signal is fed into the radar channel of the orthogonal-polarization multiplexed transmit front end and upconverted with the radar LO carrier signal to generate a horizontally polarized terahertz radar signal (220±0.85 GHz). After being amplified by the respective channel amplifiers, the terahertz communication and radar signals are fed into a 220 GHz high-isolation orthogonal-mode coupler for signal combining. The combined signal is then radiated into the wireless channel through a 220 GHz dual-polarized antenna.

At the receiver, the terahertz communication signal propagating through the wireless channel is captured by a vertically oriented single-polarization antenna and down-converted using the communication LO carrier signal. After further down-conversion by the IF down-conversion module, the signal is fed into the real-time communication baseband processing platform for demodulation. The terahertz radar signal in the wireless channel is reflected by a corner reflector and captured by a horizontally oriented single-polarization antenna located on the transmitter side. After being down-converted using the radar LO carrier signal, the signal is fed into the radar ranging baseband processing platform for data processing.

The system employs a coherent-reference architecture. For each link, a common LO source is split between the transmitter and receiver to ensure frequency synchronization and phase coherence. This enables coherent detection and accurate carrier recovery in the communication link, while preserving the phase relationship between successive radar echoes for coherent integration and improved signal-to-noise ratio. The communication and radar links use independent LO sources at the same nominal frequency. Channel isolation is achieved primarily through orthogonal polarization, polarization-selective antennas, and an OMT rather than frequency separation, while the independent LO chains minimize coupling between the two frequency-conversion paths.

A standard metallic trihedral corner reflector is used as the radar target in this study. The incident wave undergoes three successive reflections from mutually orthogonal metallic faces and is redirected toward its source while ideally retaining its linear polarization. The reflector aperture is much larger than the free-space wavelength at 220 GHz (λ ≈ 1.36 mm), and its surface roughness is much smaller than this wavelength, ensuring that specular reflection is dominant. In addition, the reflector is aligned with the boresight of the horizontally polarized radar link, while propagation-induced depolarization is expected to be negligible over the short indoor path through dry air. Therefore, the trihedral corner reflector satisfies the requirements of this study.

In practice, wall reflections—even-bounce geometries such as dihedral corners, rough surfaces, and anisotropic materials—can introduce cross-polarized components. However, unwanted cross-polarized coupling is mitigated by the polarization-selective antennas, high-isolation OMT, and antenna directivity, with propagation and material losses at 220 GHz providing additional attenuation of indirect paths. The desired co-polarized path is thus expected to dominate in the intended short-range indoor scenarios.

The real-time communication baseband processing platform was provided by the Communication Anti-Jamming Laboratory of the University of Electronic Science and Technology of China. A photograph of the platform is shown in Figure 2, and its operating principle is illustrated in Figure 3. At the transmitter, the FPGA performs high-speed channel encoding and digital modulation of the information data. A cyclic prefix and pilot symbols are then inserted, followed by a time-synchronization header, to construct the transmission frame. After digital up-conversion, the processed signal is converted into an analog waveform by a digital-to-analog converter (DAC) and output over the frequency range of 1.6 ± 0.5 GHz. At the receiver, the analog signal from the RF channel is digitized by an analog-to-digital converter (ADC). The FPGA processes the acquired samples through digital down-conversion, frame synchronization, frequency-offset estimation and correction, cyclic-prefix removal, channel estimation, and equalization. Finally, the processed signal is demodulated to recover the transmitted data.

Owing to hardware resource constraints, the channel-coding scheme specified in the standard protocol was replaced with a customized low-density parity-check (LDPC) code to meet the requirements of high-speed encoding and decoding. Soft-decision decoding is implemented using a low-complexity min-sum algorithm, which provides a suitable tradeoff between decoding performance and FPGA resource consumption. The ADC and DAC used in the baseband-processing platform are commercially available devices, and the sampling rate of each channel is set to 6.4 GSa/s. Under the current hardware configuration, the FPGA-based baseband platform supports real-time QPSK modulation, demodulation, coding, and decoding, but does not support real-time higher-order QAM processing. QPSK was therefore selected to demonstrate real-time payload-data transmission. This choice was limited by the available baseband hardware rather than by the SNR of the 220 GHz wireless link.

The bit error rate (BER) represents the ratio of incorrectly detected bits to the total number of transmitted bits and is a commonly used metric for evaluating communication performance. The relationship between BER and error vector magnitude (EVM) is expressed as follows:

(1) $$BER\approx \frac{2(1-L^{-1})}{\log_{2}L}\cdot Q\cdot \sqrt{(\frac{3\log_{2}L}{L^{2}-1})\frac{2}{EVM^{2}\cdot \log_{2}M}}$$

Here, M denotes the modulation order, and L represents the number of amplitude levels along each dimension of the constellation. For QPSK, 16QAM, and 64QAM, the modulation orders are M = 4, M = 16, and M = 64, respectively.

Based on an additive white Gaussian noise (AWGN) channel and considering the specific LDPC coding scheme implemented on the real-time communication baseband platform, the relationship between the demodulation SNR and BER for QPSK, 16QAM, and 64QAM was simulated, as shown in Figure 4.

The range resolution and the range accuracy characterize different aspects of radar performance. Range resolution describes the ability to distinguish two closely spaced targets. Using the conventional two-way delay-to-range relationship, the nominal bandwidth-limited range resolution is as follows:

(2) $$\Delta R=\frac{c}{2B_{s}}$$

Here, c is the speed of light, and Bs is the swept bandwidth of the radar waveform. For Bs = 1.7 GHz, the range resolution is approximately 8.82 cm.

Ranging accuracy is the most important metric for evaluating the performance of a radar ranging system. The ranging accuracy of an FMCW radar is primarily determined by the frequency-modulation bandwidth $B_{S}$, while the SNR of the demodulated signal also affects the accuracy of range measurement. The theoretical range-measurement accuracy $\sigma_{R}$ is related to the SNR of the demodulated signal and the frequency-modulation bandwidth $B_{S}$ as follows:

(3) $$\sigma_{R}=\frac{c}{2B_{s}}\cdot \frac{1}{\sqrt{SNR}}$$

To achieve ranging accuracy $\sigma_{R}$ = 1 cm, the SNR in the decibel scale is 18.9 dB. Therefore, under the approximate model adopted herein, when the effective SNR at the output of the matched filter, or the effective SNR associated with peak estimation, is approximately 19 dB, the theoretical standard deviation of the random ranging error is approximately 1 cm.

In our system, an LFM baseband signal with a bandwidth of 1.7 GHz is employed. The echo signal is acquired using a high-speed oscilloscope (DSO91304A), which supports a maximum sampling rate of 40 GSa/s. This sampling rate is sufficient for acquiring the 1.7 GHz-bandwidth echo signal in accordance with the Nyquist sampling theorem.

In Figure 5, the range-domain matched-filter function is given by the following:

(4) $$H(f_{t_{r}})=rect\left\{\frac{f_{t_{r}}}{\left|K\right|T}\right\}\exp(j\pi \frac{f_{t_{r}}^{2}}{K_{t_{r}}})$$

The time-domain waveform of the baseband signal is shown in Figure 6. The sampling rate is 20 GSa/s, the number of samples is Nt = 768, and the signal duration is T = 38.4 ns.

By applying range-domain matched filtering and the fractional Fourier transform (FRFT), the signal-to-noise ratio (SNR) of the baseband echo signal output by the ADC can be increased to above 20 dB, thereby enabling a ranging precision better than 1 cm.

For the 220 GHz orthogonal-polarization multiplexing radar channel used in this paper, the modulation bandwidth is 1.7 GHz. To achieve centimeter-level ranging accuracy, the SNR of the demodulated signal must be greater than 19 dB. Since the radar signal received at the receiver is an echo reflected by a corner reflector, the transmission distance of the radar signal is twice that of the communication signal, resulting in a 6 dB higher path loss. Therefore, if the SNR of the demodulated radar signal is greater than 19 dB, the SNR of the demodulated communication signal should be greater than 24 dB, enabling a BER on the order of $10^{-8}$.

Under an ambient temperature of 25 °C, an atmospheric pressure of 101.3 kPa, and a humidity of 70%, the atmospheric attenuation of a 220 GHz wireless signal is 2.35 dB/km. Based on the measured conversion gain of the transmit chain and the noise figure of the receive chain, and taking into account factors such as the experimental environment, the final wireless communication distance of the system is determined to be 0.25 m. The specific link budget parameters are listed in Table 1.

## 3. Key Circuit Components and Performance

The proposed 220 GHz THz integrated sensing and communication (ISAC) system comprises several key modules, including a dual-polarization transmit front end with a dual-polarized antenna, two single-polarization receive front ends with corresponding antennas, a real-time communication baseband-processing platform, an intermediate-frequency (IF) conversion module, a radar-ranging baseband-processing platform, and a corner reflector. The main components and their measured performance are described below.

Many existing wireless communication systems employ frequency-division multiplexing (FDM) to transmit multiple signals over separate frequency bands. In an FDM system, channel separation is achieved in the frequency domain, and sufficiently selective filters or guard bands may be required to suppress inter-channel interference. In contrast, orthogonal-polarization multiplexing (OPM) enables multiple signals to share the same frequency band by assigning them to mutually orthogonal polarization states. Figure 7 is provided for comparison between FDM and OPM; the proposed ISAC system adopts OPM as its multiplexing scheme.

At THz frequencies, dielectric materials may introduce substantial transmission loss. Metallic waveguides are therefore preferred for the OMT to reduce dielectric loss and maintain low-loss signal transmission. For an OMT whose common port is a square waveguide (SWG), the two orthogonally polarized signals are supported by the two degenerate fundamental modes, conventionally denoted as $TE_{10}$ and $TE_{01}$. According to the coordinate definition used in this work, these two modes correspond to the vertically and horizontally polarized electric fields, respectively.

Because the OMT contains several geometrical discontinuities, higher-order modes may be excited along the waveguide transmission path, particularly near the coupling and branching regions. The total electromagnetic field inside the waveguide, denoted by $EM_{w}$, can be expressed as follows:

(5) $$EM_{w}=\sum_{p=1}^{\infty }TE_{p0}+\sum_{q=1}^{\infty }TE_{0q}+\sum_{p=1}^{\infty }\sum_{q=1}^{\infty }TE_{pq}+\sum_{p=1}^{\infty }\sum_{q=1}^{\infty }TM_{pq}$$

where the terms represent the fundamental and higher-order modal components supported by the waveguide.

If the discontinuities are symmetric with respect to the plane associated with the vertical polarization, the structural symmetry imposes selection rules on the excited modes. Modes with incompatible field symmetry cannot be excited, and the total field associated with the vertical-polarization path can therefore be expressed as follows:

(6) $$EM_{v}=\sum_{p=1}^{\infty }TE_{(2p-1)0}+\sum_{q=1}^{\infty }TE_{0q}+\sum_{p=1}^{\infty }\sum_{q=1}^{\infty }TE_{(2p-1)q}+\sum_{p=1}^{\infty }\sum_{q=1}^{\infty }TM_{(2p-1)q}$$

Similarly, symmetry with respect to the plane associated with the horizontal polarization suppresses modes with incompatible field symmetry. The corresponding total field can be written as follows:

(7) $$EM_{h}=\sum_{p=1}^{\infty }TE_{p0}+\sum_{q=1}^{\infty }TE_{0(2q-1)}+\sum_{p=1}^{\infty }\sum_{q=1}^{\infty }TE_{p(2q-1)}+\sum_{p=1}^{\infty }\sum_{q=1}^{\infty }TM_{p(2q-1)}$$

The excitation of higher-order modes can significantly reduce the usable single-mode bandwidth of the dominant mode. Therefore, maintaining appropriate symmetry with respect to both polarization planes is important for suppressing unwanted mode conversion and extending the usable bandwidth of the OMT. In practice, this structural symmetry must be combined with optimized impedance matching and coupling transitions to achieve broadband operation.

The operating principle of the proposed dual-symmetric OMT is illustrated in Figure 8. Two types of double-ridge sections are introduced into the SWG, forming a double-inner-ridge waveguide (DIWG) and a double-outer-ridge waveguide (DOWG), respectively. The dual-symmetric reverse-coupling section consists of the DIWG, the DOWG, and a double-ridge coupling plate (DRCP). The horizontally polarized (HP) signal is separated from the vertically polarized (VP) signal through the coupling between the DIWG and the DRCP. The VP signal is separated from the HP signal through the connecting structure formed by the DIWG and DOWG.

The complete structure of the proposed high-isolation WR-4.3 OMT (Chengdu Xixia Technology Development Co., Ltd., Chengdu, China), designed to cover 170–260 GHz, is shown in Figure 9. The OMT consists of a dual-symmetric reverse-coupling section, a Y-junction, two 180° E-plane bends, and a 90° H-plane tapered transition. The HP signal is separated from the VP signal through the coupling structure formed by the DIWG and DRCP and is then routed to Port 2 through the 180° E-plane bends and the Y-junction. The VP signal is separated from the HP signal through the connecting structure formed by the DIWG and DOWG and is then routed to Port 3 through the 90° H-plane tapered transition.

Each component was first simulated separately, after which the complete OMT was jointly optimized to achieve the target performance. The finite conductivity of the metal used in fabrication was included in the simulations. The simulated performance of the optimized OMT is shown in Figure 10. Over the 170–260 GHz frequency range, the HP path exhibits a return loss better than 20 dB and an insertion loss below 0.45 dB, with average values of 25.5 and 0.36 dB, respectively. The VP path exhibits a return loss better than 20 dB and an insertion loss below 0.2 dB, with average values of 24.1 and 0.13 dB, respectively. The simulated isolation between the HP and VP paths exceeds 62.5 dB over the entire band, with an average value of 68 dB. These results indicate that the proposed OMT provides broadband impedance matching, low insertion loss, and high isolation between the two orthogonal polarization paths.

To experimentally verify the design, a WR-4.3 OMT was fabricated and measured over the 170–260 GHz frequency range. During the stand-alone OMT measurements, the ports under test were connected to the measurement ports of a vector network analyzer (VNA), while the ports not under test were terminated with matched loads. The measured results are shown in Figure 11. Over this frequency range, the HP path exhibits an average return loss of 24 dB, with a minimum value of 15 dB, and an average insertion loss of 0.6 dB, with a maximum value of 1.2 dB. The VP path exhibits an average return loss of 24 dB, with a minimum value of 17.5 dB, and an average insertion loss of 0.5 dB, with a maximum value of 1.1 dB. The measured return loss and insertion loss are in reasonable agreement with the simulated results. The measured isolation between the two polarization paths averages 54 dB, with a minimum value of 46 dB. The discrepancy between the measured and simulated isolation is mainly attributed to fabrication and assembly tolerances at the interface between the two OMT halves. These tolerances may disturb the intended structural symmetry and coupling balance, thereby degrading the measured isolation.

During the second frequency-conversion stage of the polarization-multiplexed communication link, a 220 GHz subharmonic mixer is employed. The mixer is constructed using domestically produced anti-parallel Schottky diodes. Through subharmonic mixing, the 5.4 GHz signal output by the intermediate-frequency IQ module is upconverted to the terahertz frequency band. Since the mixer’s conversion loss is one of the key performance indicators affecting the transmit signal power of the communication system, the conversion loss of the mixer was measured. The mixer prototype and the measurement results are shown in Figure 12. As indicated by the measurement results, the mixer exhibits a conversion loss of less than 10 dB over an RF frequency range of 190–235 GHz.

In the transmit chain, the two orthogonally polarized signals combined by the OMT are radiated into the wireless channel through a 220 GHz dual-polarized antenna. A photograph of the antenna and its measured performance are shown in Figure 13. At 220 GHz, the antenna provides a gain of 14 dBi, and its measured polarization isolation exceeds 45 dB over the angular range from −50° to +50°. These results characterize the gain and polarization-separation performance of the dual-polarized transmit antenna.

The 220 GHz OPM transmit front end consists of two independent channels, as shown in Figure 14. The two channels transmit the communication and radar signals using vertical and horizontal polarization, respectively. Each channel is implemented using discrete components, including a 220 GHz power amplifier, a 220 GHz subharmonic mixer, and a 110 GHz frequency-octupler chain. A Ku-band signal from the signal source is first applied to the LO frequency-octupler chain, which generates a nominal 107.3 GHz LO signal. The LO signal then drives the subharmonic mixer, where it is combined with the corresponding modulated 5.4 GHz IF signal to generate a 220 GHz THz carrier carrying the modulation information. The generated THz signal is subsequently amplified by the 220 GHz power amplifier. Finally, the two orthogonally polarized signals are combined through the high-isolation OMT.

A test platform was set up to measure the polarization isolation between the two channels of the assembled transmit front end, and the results are shown in Figure 14b. The measured polarization isolation exceeds 25 dB over the frequency range of 204–222 GHz. This front-end-level result should be distinguished from the stand-alone OMT isolation exceeding 45 dB shown in Figure 11c, because the two measurements involve different external loading and integration conditions. In the complete dual-polarization transmit front end, impedance mismatches, interface reflections, nonideal waveguide joints and transitions, and the finite cross-polarization discrimination of the antenna can introduce additional mode conversion and inter-channel coupling, thereby reducing the front-end-level isolation. Despite this reduction, the complete dual-polarization transmit front end maintains a polarization isolation of more than 25 dB over 204–222 GHz, and the system can still support simultaneous communication and radar ranging. Given the closely matched conversion gains of the two channels near 220 GHz, 220 GHz was selected as the center frequency of the transmit front end.

As shown in Figure 15, the single-polarization receiver front end consists of a 220 GHz subharmonic mixer, a 110 GHz frequency-octupler chain, and a standard-gain horn antenna. The standard-gain horn antenna provides a gain of 25 dBi at 220 GHz, thereby increasing the received signal power and relaxing the required sensitivity of the receiver front end.

The noise figure of the receiver front end was measured using the Y-factor method, as shown in Figure 16. The receiver front end was characterized under two thermal conditions: 295 K and 357 K. The corresponding output powers were measured using a spectrum analyzer. The Y-factor was obtained from the ratio of the two measured output powers, and the equivalent noise temperature and noise figure were then calculated accordingly. The measured noise figure is below 11 dB over the 204–222 GHz frequency range and is 9.8 dB at 220 GHz.

## 4. Experimental Demonstration of a 220 GHz Orthogonal-Polarization-Multiplexed ISAC System

To experimentally verify the proposed 220 GHz integrated sensing and communication (ISAC) system, a wireless transmission link was established using the orthogonal-polarization multiplexing (OPM) architecture. Unlike frequency-division multiplexing (FDM), which separates signals using different frequency bands, the proposed system transmits the communication and radar signals within the same 220 GHz frequency band using two orthogonal polarization states. The communication signal is transmitted through the vertically polarized channel, whereas the radar signal is transmitted through the horizontally polarized channel.

First, the transmit and receive baseband units were tested to verify the operation of the real-time communication baseband-processing platform. The constellation diagram of the demodulated signal exhibits no apparent rotation or angular displacement, and the constellation points are well concentrated around their reference positions, as shown in Figure 17. These results indicate that the baseband loopback signal has a well-defined constellation with limited noise and residual frequency offset. The loopback test yielded an SNR of 32.71 dB, an EVM of 2.31%, and a BER of $2.88\times 10^{-8}$, indicating that the baseband platform satisfies the communication requirements.

A complete 220 GHz ISAC wireless transmission link was subsequently established, as shown in Figure 18. The left-hand terminal was used for communication reception and was equipped with a vertically polarized 220 GHz receiver front end. A pyramidal corner reflector was placed in the radar path to reflect the horizontally polarized radar signal. The right-hand terminal was used for radar reception and was equipped with a horizontally polarized 220 GHz receiver front end. The separation between the two terminals was fixed at 0.25 m. The radar-target distance was adjusted by changing the position of the corner reflector relative to the radar receiver. The target used in the experiment is a standard trihedral corner reflector, which ideally preserves the polarization state of the incident wave. The echo therefore remains predominantly co-polarized with the transmitted horizontally polarized radar signal, and the horizontally polarized receiver mainly detects the co-polar component. Therefore, the communication-link distance and the radar-target distance were defined independently.

To evaluate the simultaneous communication and radar-ranging capability of the proposed system, experiments were conducted under the following three conditions:

1. No pyramidal corner reflector was placed in the radar path.
2. The pyramidal corner reflector was positioned 0.25 m from the radar receiver.
3. The pyramidal corner reflector was positioned 0.4 m from the radar receiver.

The communication-link distance was maintained at 0.25 m under all three conditions. In addition, the programmable attenuation of the IF-conversion module was adjusted so that the integrated power of the modulated IF signal at the transmitter was maintained at −10 dBm for all measurements. The communication and radar signals were generated using the 5.4 GHz IF architecture described in the preceding sections.

### 4.1. Communication and Radar Measurements Without a Corner Reflector

The experimental configuration without a pyramidal corner reflector is shown in Figure 19. In this configuration, the radar path was terminated with absorbing material to suppress unwanted reflections. The measured spectrum and constellation diagram of the demodulated communication signal are shown in Figure 20. The integrated power of the demodulated communication signal was −10.9 dBm. The constellation diagram exhibits no apparent rotation or angular displacement, and the constellation points are concentrated around their reference positions. The demodulated signal achieved an SNR of 24.38 dB, an EVM of 6.04%, and a BER of $2.56\times 10^{-8}$. These results demonstrate 2-Gbit/s wireless communication over a 0.25 m link under the no-target condition.

Under this condition, no intentional radar echo was generated because the radar signal was absorbed by the absorbing material. The spectrum of the demodulated radar signal and the corresponding ranging profile are shown in Figure 21. No distinct FMCW echo component is observed in the demodulated spectrum. In addition, the ranging profile has a maximum amplitude of only 1.48, indicating that no radar target was detectable under the measurement conditions. This result is consistent with the experimental configuration.

### 4.2. Communication and Radar Measurements with a Corner Reflector at 0.25 m

The experimental configuration with the pyramidal corner reflector positioned 0.25 m from the radar receiver is shown in Figure 22. The measured spectrum and constellation diagram of the demodulated communication signal are presented in Figure 23. The integrated power of the demodulated communication signal was −10.97 dBm. The constellation points were concentrated around their reference positions, with no apparent rotation or angular displacement. The demodulated signal achieved an SNR of 24.55 dB, an EVM of 5.92%, and a BER of $2.79\times 10^{-8}$. These results indicate that the introduction of the corner reflector did not cause a significant degradation in the communication link.

The radar signal was reflected by the corner reflector, and the resulting echo was captured by the horizontally polarized radar receiver. The spectrum of the demodulated radar signal and the corresponding ranging profile are shown in Figure 24. A distinct FMCW echo component is observed in the demodulated spectrum. The ranging profile exhibits a peak amplitude of 18.1, confirming the detection of the corner reflector. The detected target distance is 0.25 m, which agrees with the actual reflector position within the range-measurement accuracy.

### 4.3. Communication and Radar Measurements with a Corner Reflector at 0.4 m

The experimental configuration with the pyramidal corner reflector positioned 0.4 m from the radar receiver is shown in Figure 25. The measured spectrum and constellation diagram of the demodulated communication signal are shown in Figure 26. The integrated power of the demodulated communication signal was −10 dBm. The constellation diagram exhibits no apparent rotation or angular displacement, and the constellation points remain concentrated around their reference positions. The demodulated signal achieved an SNR of 24.06 dB, an EVM of 6.26%, and a BER of $4.66\times 10^{-8}$. These results demonstrate stable 2-Gbit/s data transmission despite the increased radar-target distance.

The radar signal was reflected by the corner reflector and captured by the horizontally polarized radar receiver. The demodulated radar spectrum and ranging profile are shown in Figure 27. A distinct FMCW echo component remains observable in the demodulated spectrum. Because the radar-target distance increased, the received echo power decreased, as indicated by the reduction in the ranging-profile peak. The peak amplitude was 11.3. The measured target distance was 0.39 m, compared with the actual distance of 0.4 m. The resulting ranging error was 1 cm.

The three measurement conditions also allow the effect of the radar echo on the communication link to be assessed directly. The demodulated communication signal exhibits an SNR of 24.38 dB and an EVM of 6.04% without the corner reflector, 24.55 dB and 5.92% with the reflector at 0.25 m, and 24.06 dB and 6.26% with the reflector at 0.40 m, with the BER remaining at the same order of magnitude in all three cases. No systematic degradation of the communication link is observed when the corner reflector is introduced, and the small variations among the three conditions are comparable to the measurement repeatability. These results indicate that, for the non-depolarizing trihedral target used here, the energy of the cross-polarized radar leakage into the communication channel is negligibly small and therefore does not appreciably degrade system performance.

Table 2 compares the proposed system with related terahertz wireless communication systems reported in the literature. The systems listed in the comparison mainly focus on high-speed communication, whereas the proposed system additionally supports radar ranging. By using OPM, the proposed 220 GHz system transmits the communication and radar signals within the same frequency band and separates them through orthogonal polarization. The experimental results demonstrate 2-Gbit/s wireless communication over a 0.25 m distance and a maximum radar-ranging error of 1 cm.

According to the SNR–BER relationship in Figure 4, the measured end-to-end wireless-link SNR of 24.06–24.55 dB is sufficient for 64QAM. With an AWG-based waveform and higher-speed processing, the combined increase in modulation order and symbol rate could potentially increase the nominal data rate by up to eight times compared with the present 2 Gbit/s QPSK implementation, provided that sufficient SNR is maintained.

## 5. Conclusions

A 220 GHz integrated sensing and communication system based on orthogonal-polarization multiplexing was designed, fabricated, and experimentally demonstrated. The proposed system uses orthogonal polarization, rather than frequency-domain separation, to simultaneously support communication and radar ranging within the same frequency band.

First, a dual-symmetric reverse-coupled OMT was developed for polarization separation and combination. The fabricated OMT operates from 170 to 260 GHz. The measured minimum return losses are 15 and 17.5 dB for the HP and VP channels, respectively, and the measured polarization isolation exceeds 45 dB over the operating band. The design adopts a dimensional-tolerance requirement of approximately 10 μm, making it suitable for THz waveguide implementation.

Second, the OMT was integrated with solid-state frequency-conversion circuits, amplifiers, waveguide transitions, and a dual-polarized antenna to construct the 220 GHz dual-polarization transceiver front end. The 5.4 GHz IF signal and 107.3 GHz LO signal are combined in a subharmonic mixer to generate the 220 GHz carrier. The measured polarization isolation of the transmit front end is better than 25 dB over 204–222 GHz. The receive front end provides a noise figure below 11 dB over this frequency range and 9.8 dB at 220 GHz.

Finally, a wireless ISAC experiment was conducted using coherent-reference signal generation and real-time baseband processing. The system achieved 2 Gbit/s wireless communication over a 0.25 m distance with a BER below $5\times 10^{-8}$. Radar-ranging experiments were conducted with target distances of 0.25 and 0.4 m, and the maximum measured ranging error was 1 cm. These results demonstrate the feasibility of OPM for jointly supporting high-speed THz communication and centimeter-level radar ranging.

The proposed system provides an experimental demonstration of a 220 GHz OPM-based ISAC architecture. Future work will address the improvement of output power, transmission distance, data rate, polarization isolation, and front-end integration.

## Figures and Tables

**Figure 1:**
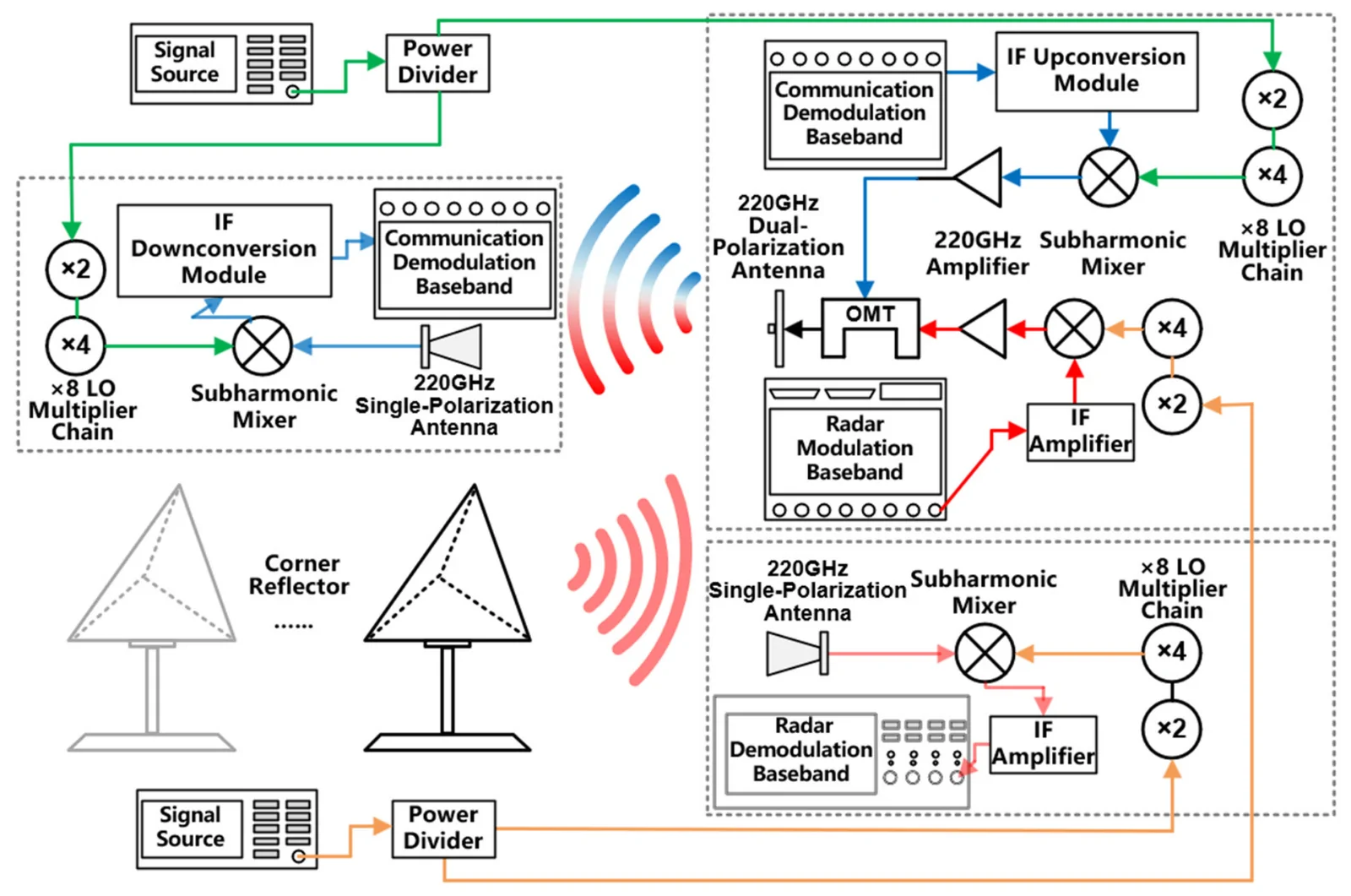
Overall architecture of the proposed 220 GHz ISAC system.

**Figure 2:**
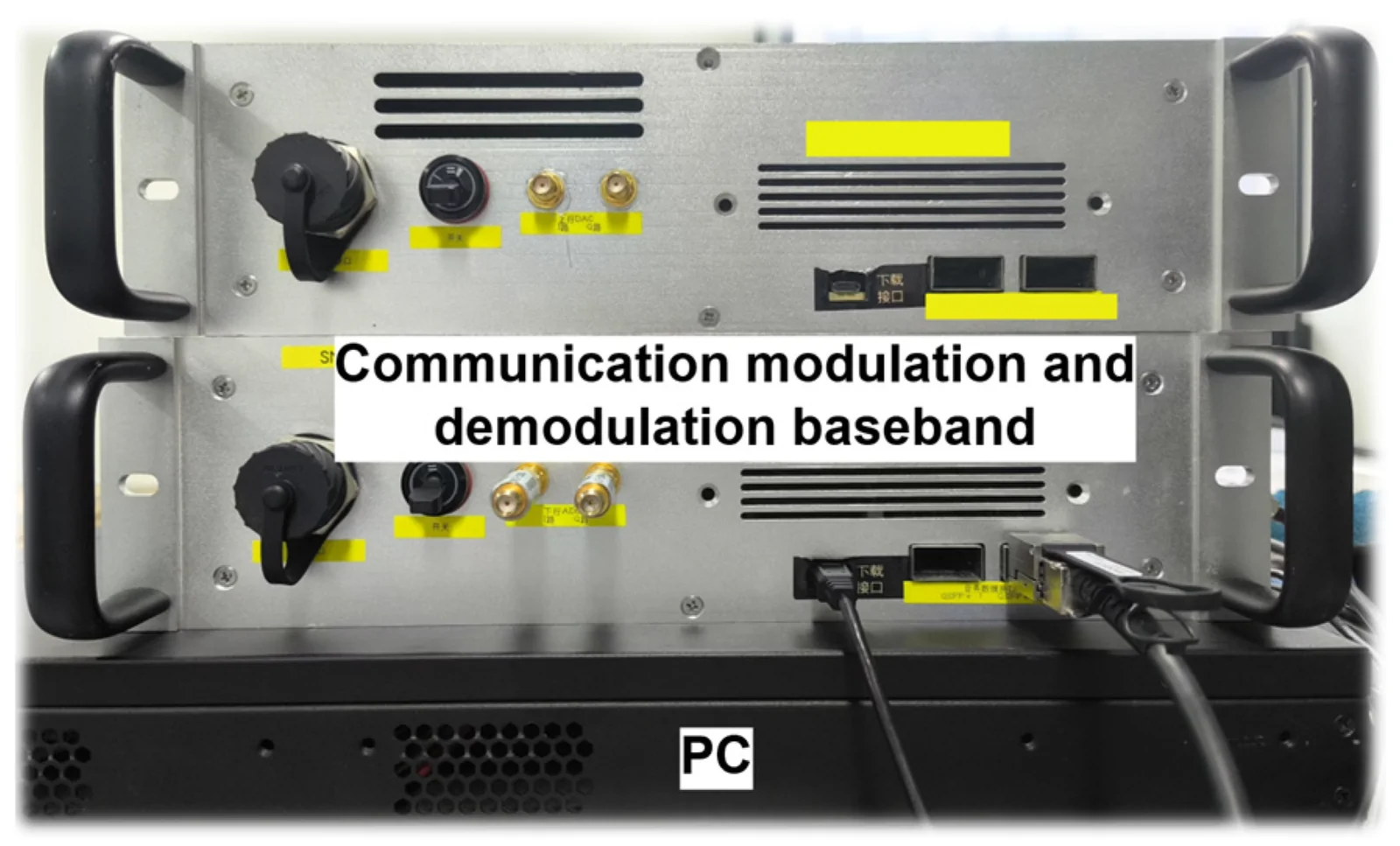
Photograph of the real-time communication baseband processing platform.

**Figure 3:**
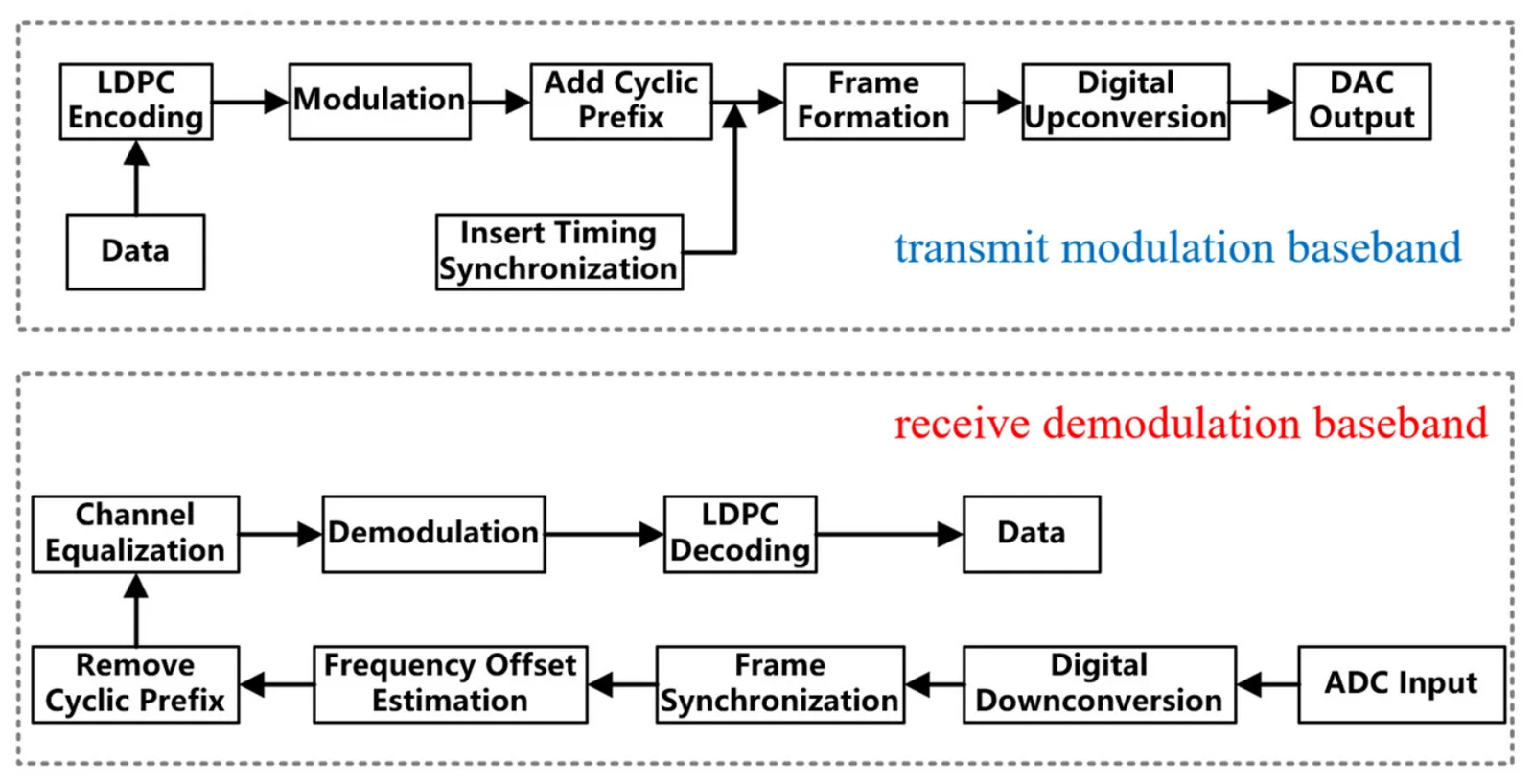
Operating principle of the real-time communication baseband processing platform.

**Figure 4:**
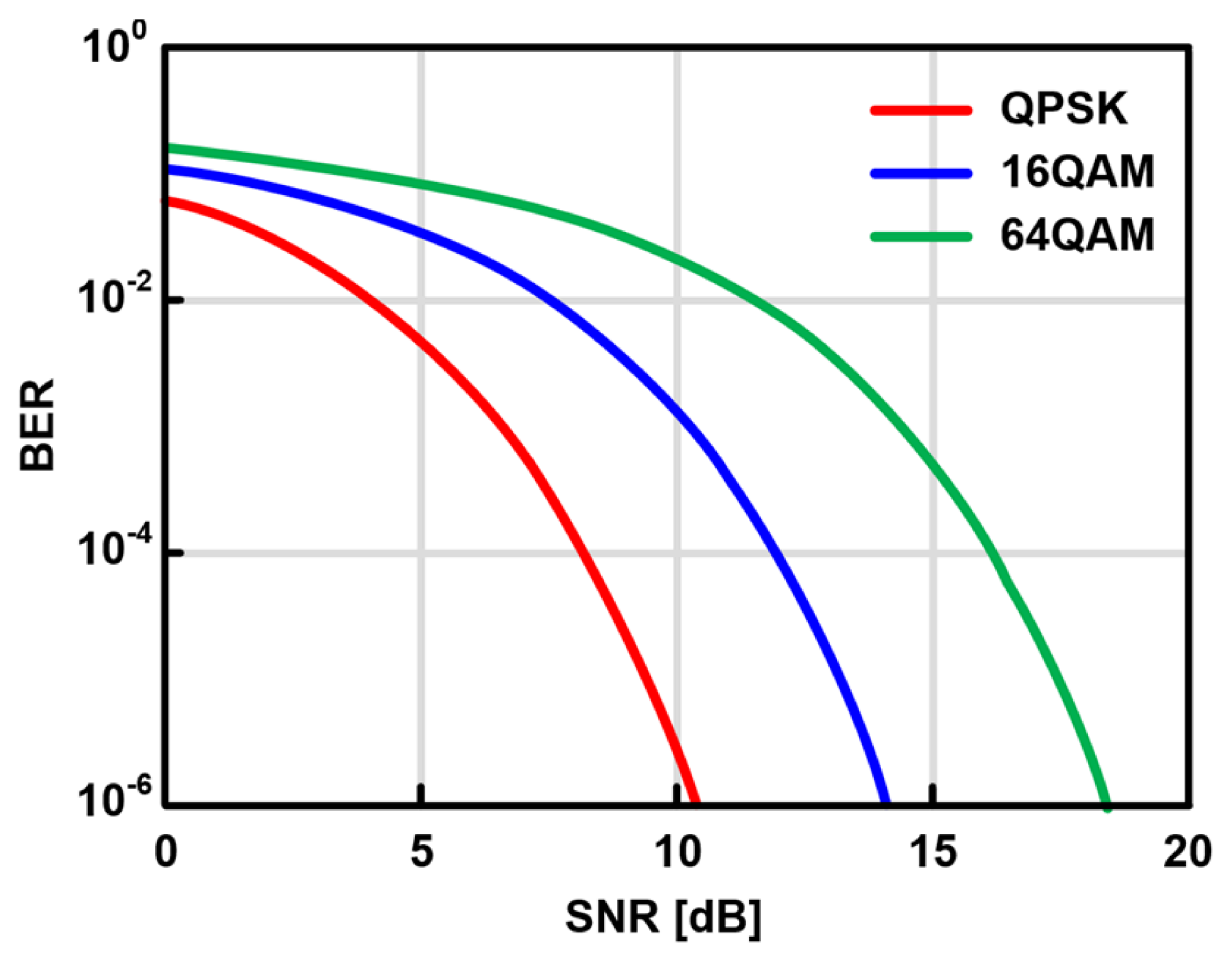
BER and required demodulation SNR for different modulation schemes.

**Figure 5:**
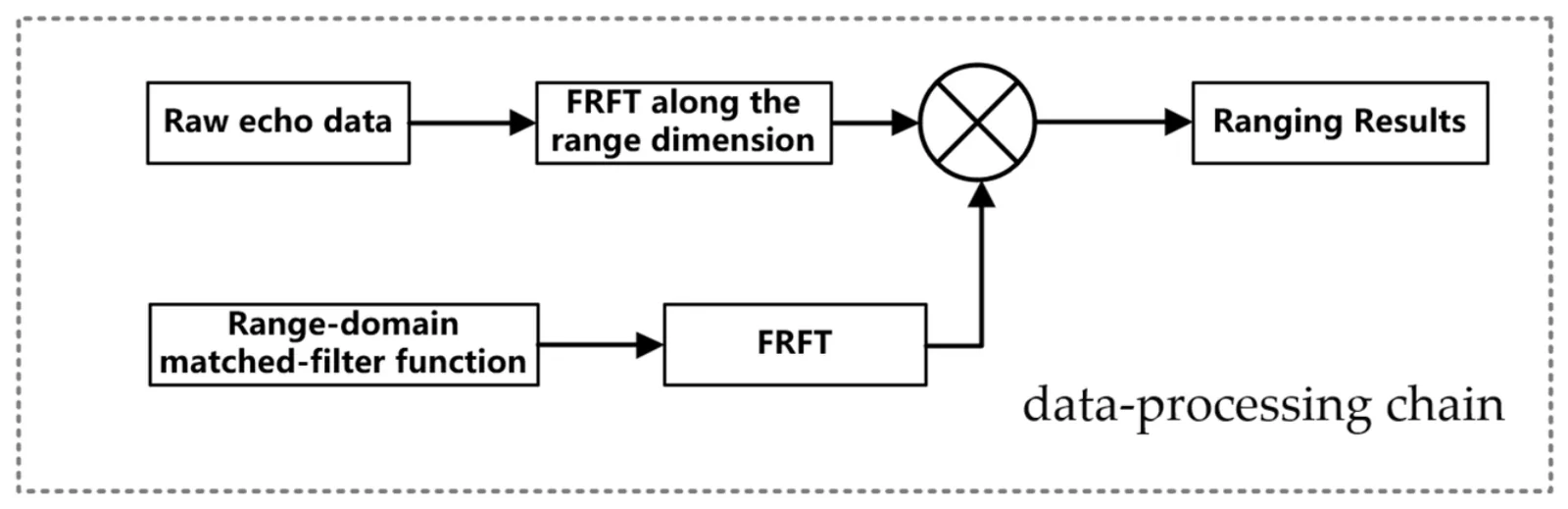
The data-processing chain of the echo signal.

**Figure 6:**
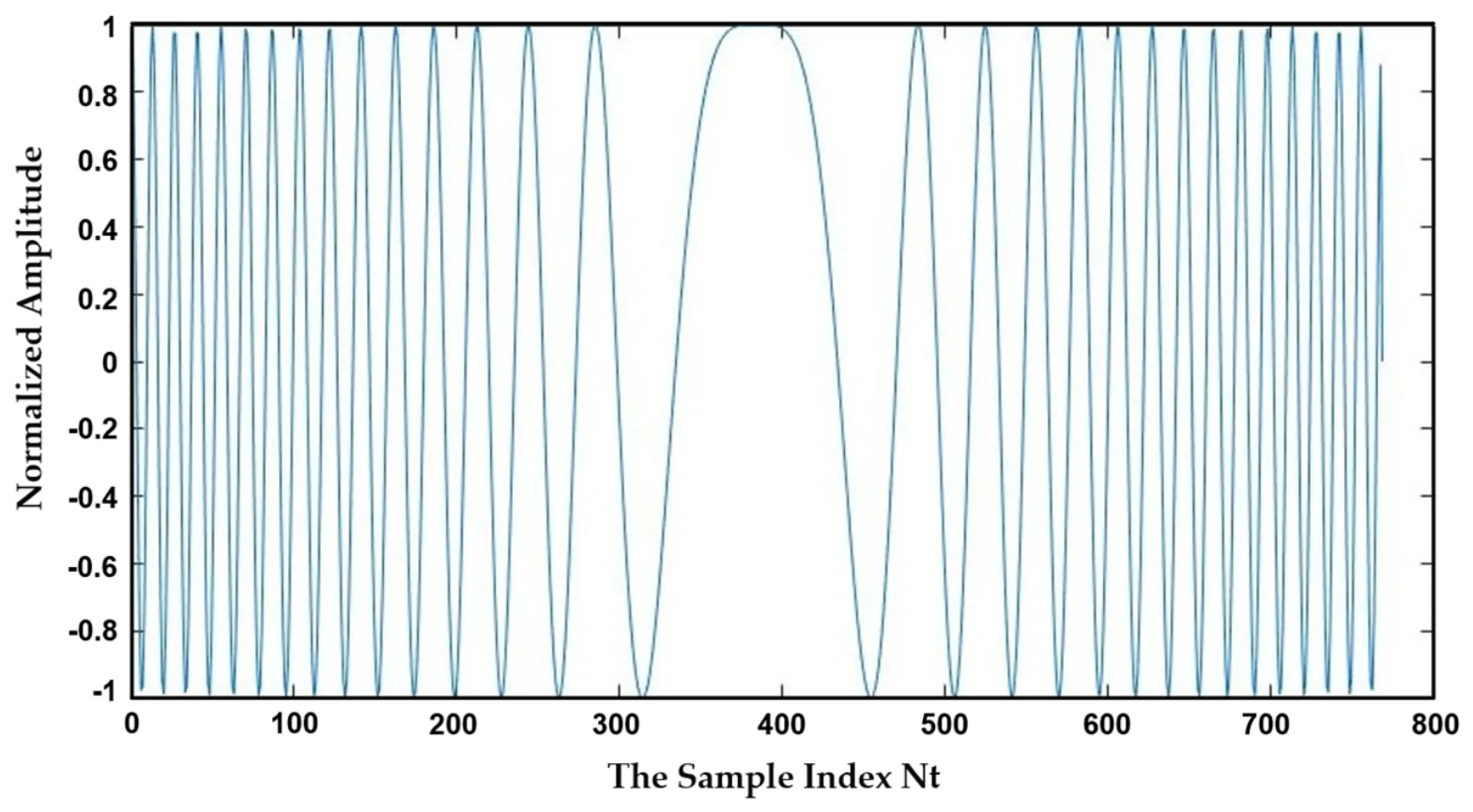
The time-domain waveform of the baseband signal.

**Figure 7:**
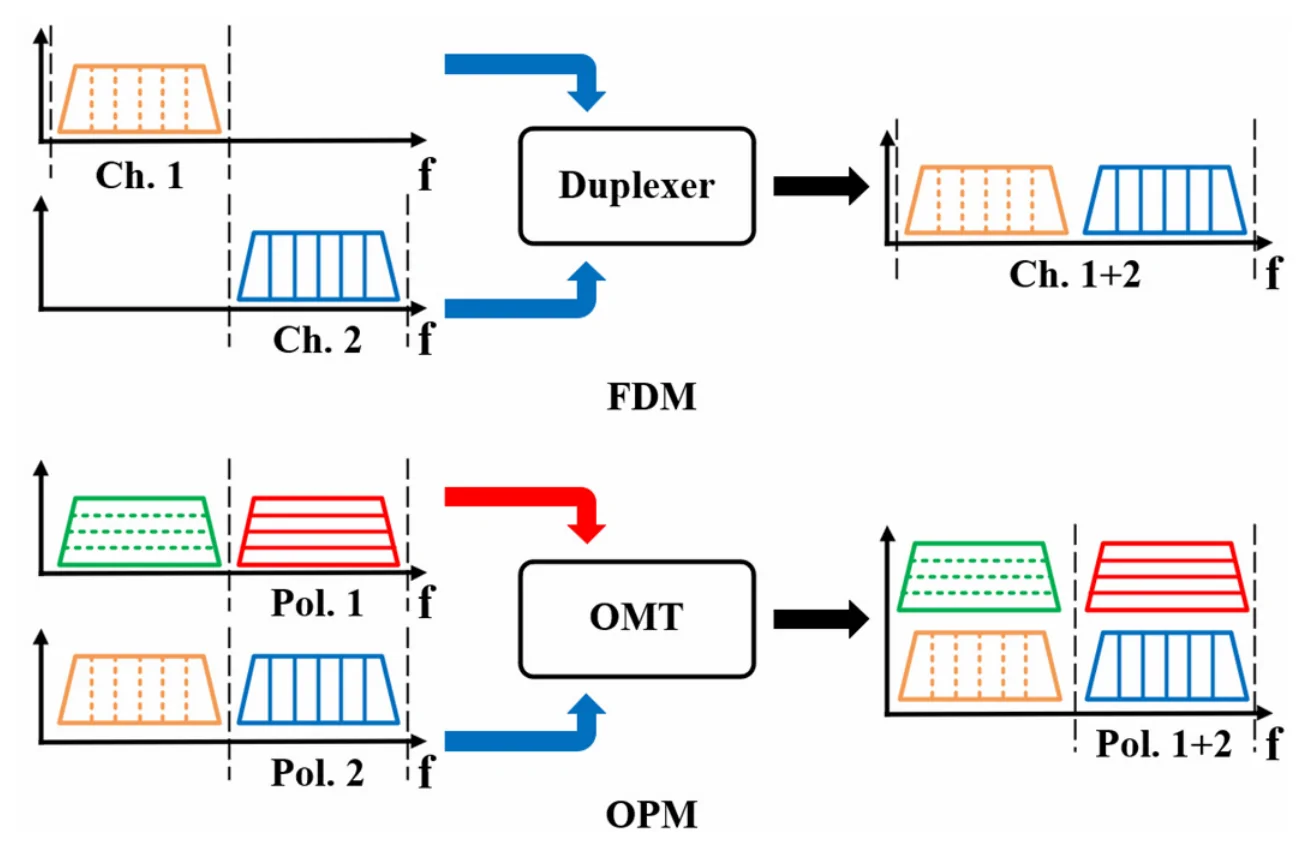
Comparison of common wireless communication systems between FDM and OPM.

**Figure 8:**
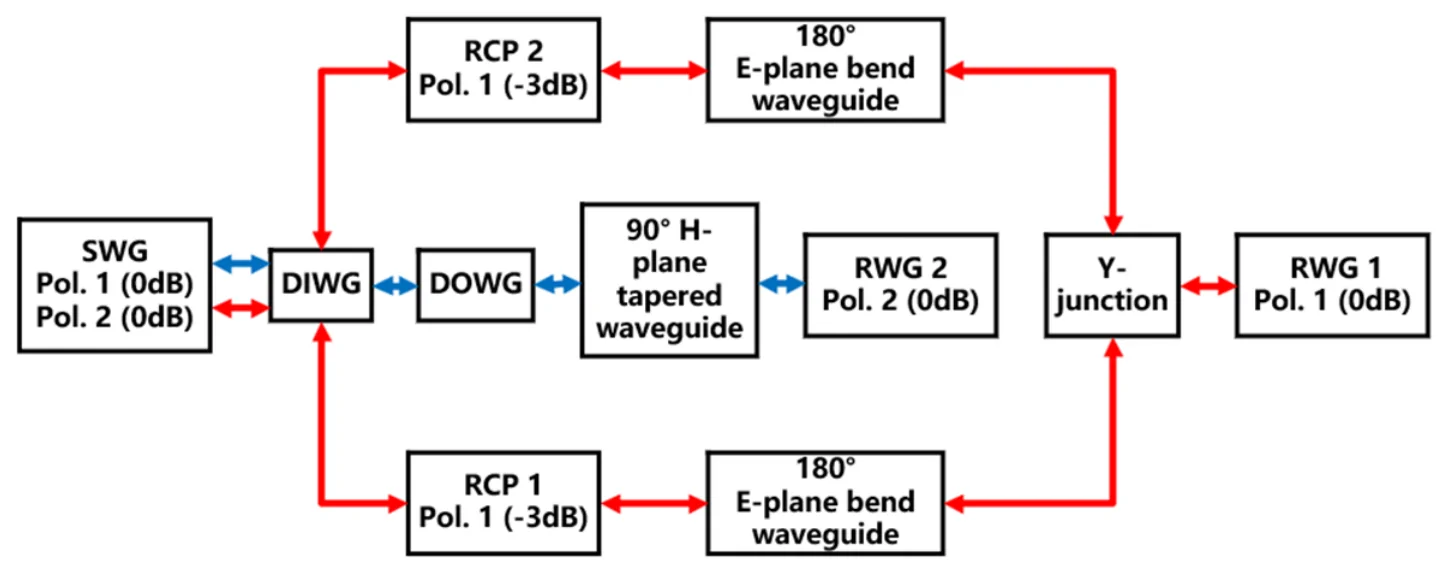
Operating principle of the proposed dual-symmetric OMT.

**Figure 9:**
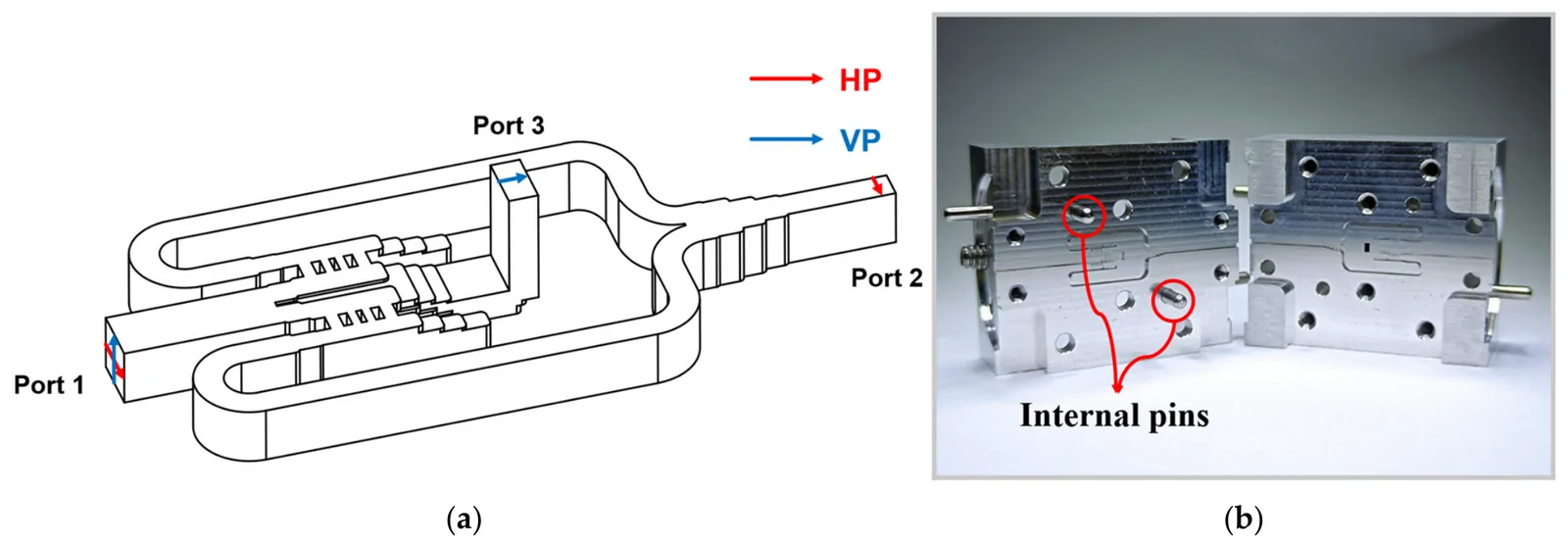
Fabricated high-isolation WR-4.3 OMT. (**a**) Three-dimensional structure. (**b**) Photograph.

**Figure 10:**
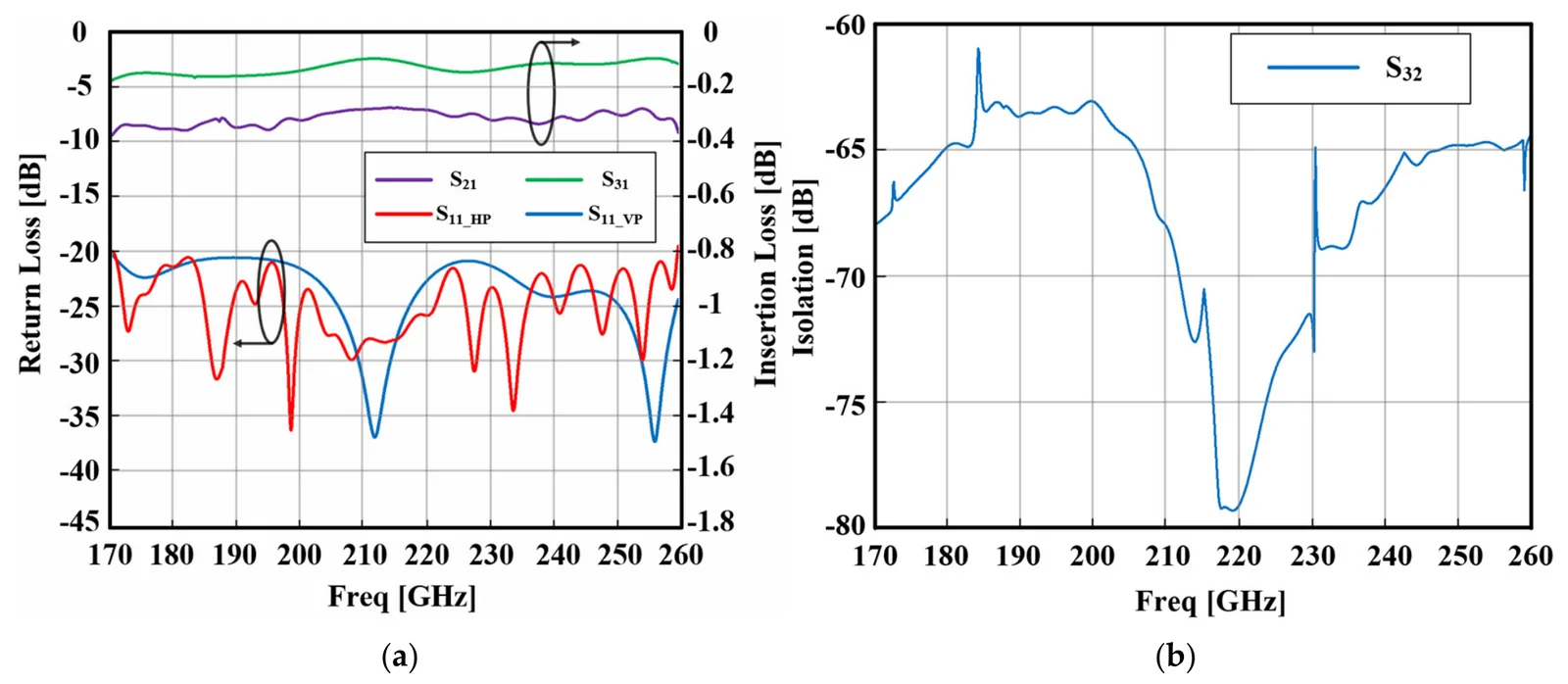
Simulated performance of the wideband, high-isolation WR-4.3 OMT. (**a**) Return loss and insertion loss. (**b**) Isolation.

**Figure 11:**
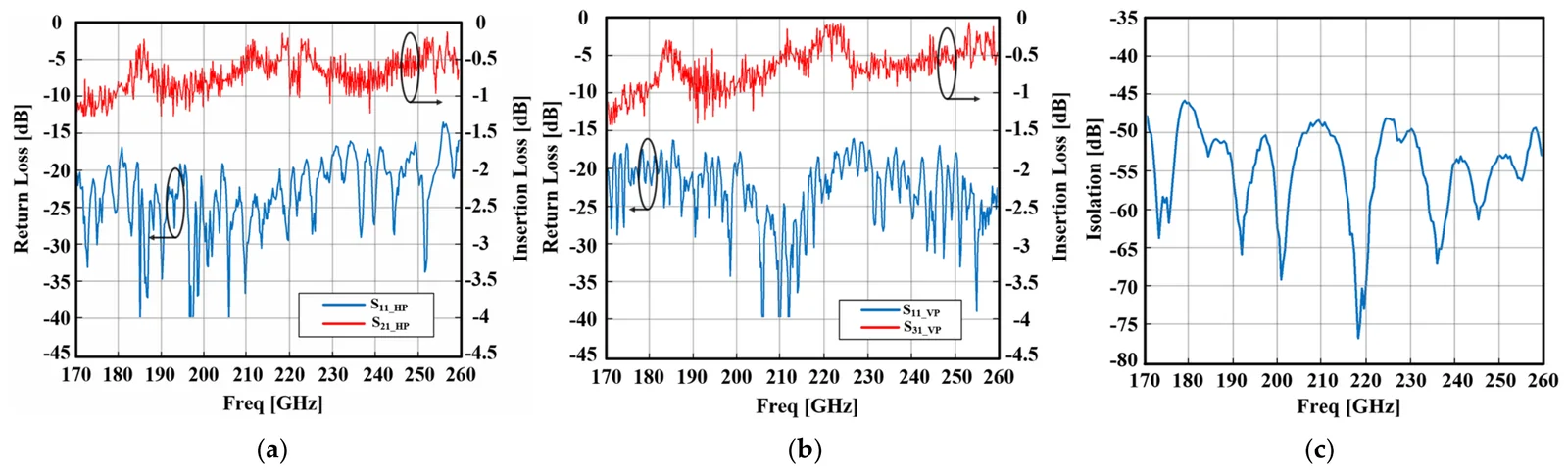
Measured performance of the fabricated OMT. (**a**) Horizontally polarized wave. (**b**) Vertically polarized wave. (**c**) Polarization isolation.

**Figure 12:**
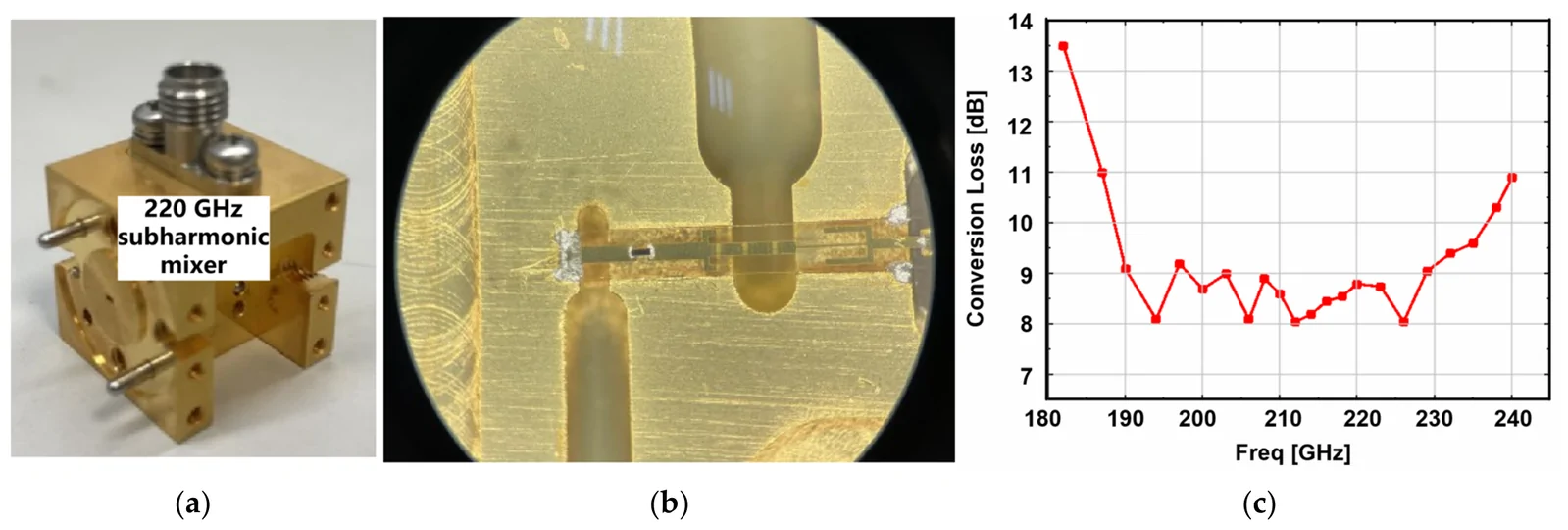
A 220 GHz subharmonic mixer. (**a**) Photograph. (**b**) Internal circuit. (**c**) Measured conversion loss.

**Figure 13:**
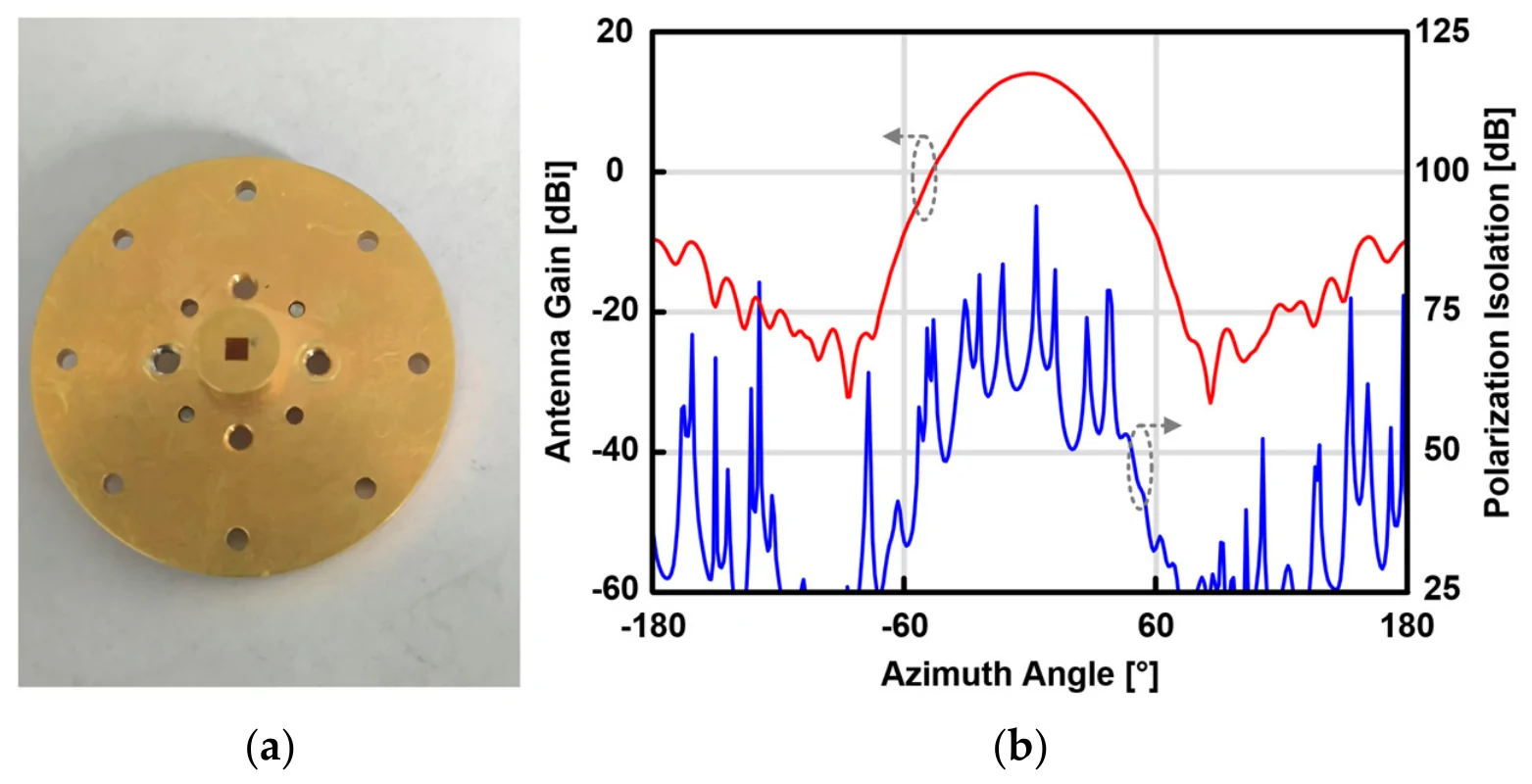
A 220 GHz dual-polarized antenna. (**a**) Photograph. (**b**) Measured performance.

**Figure 14:**
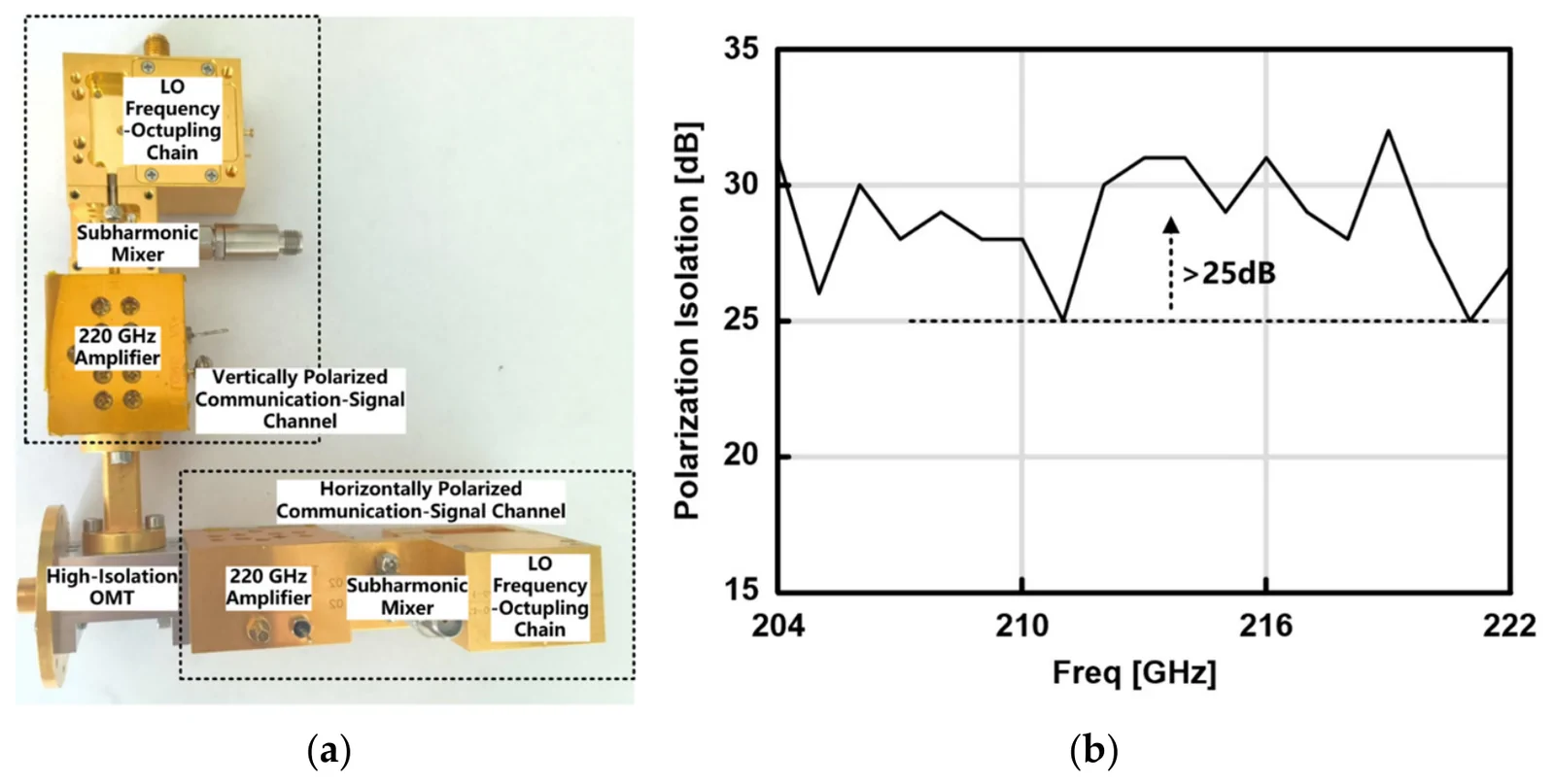
The 220 GHz dual-polarization transmit front end. (**a**) Photograph. (**b**) Measured isolation.

**Figure 15:**
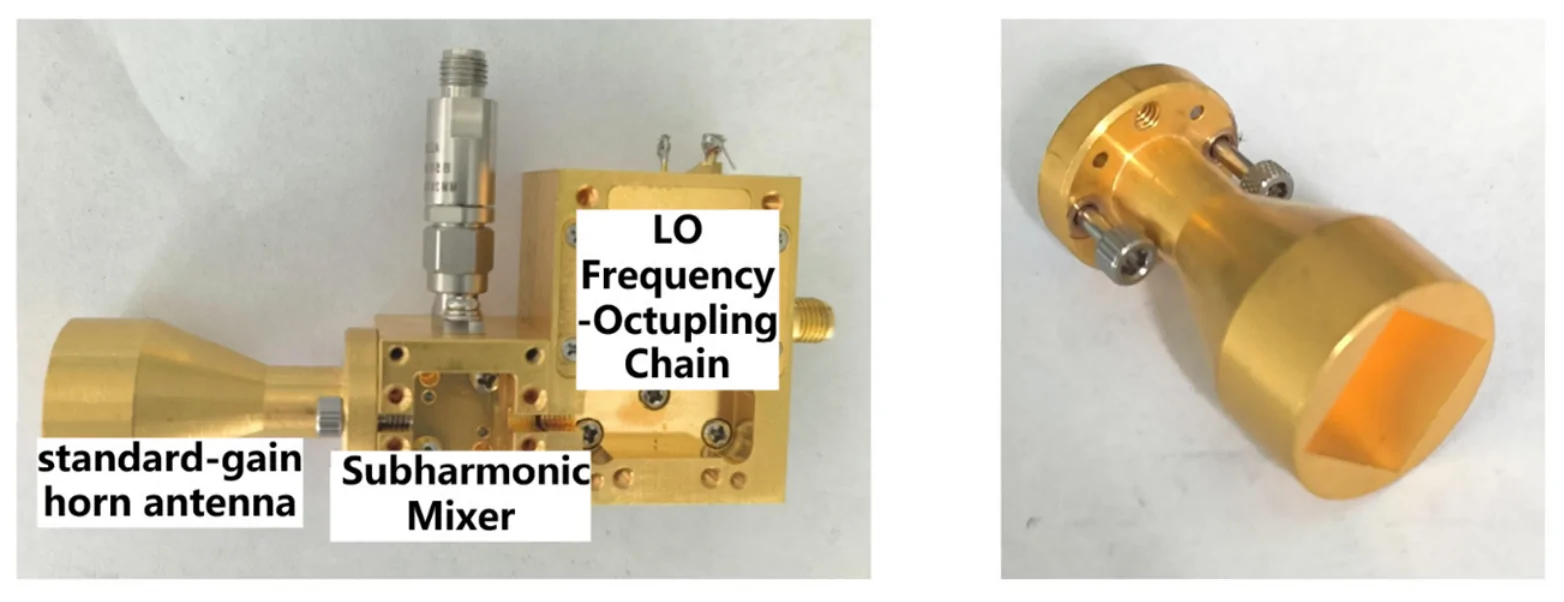
Photographs of the 220 GHz single-polarization receive front end.

**Figure 16:**
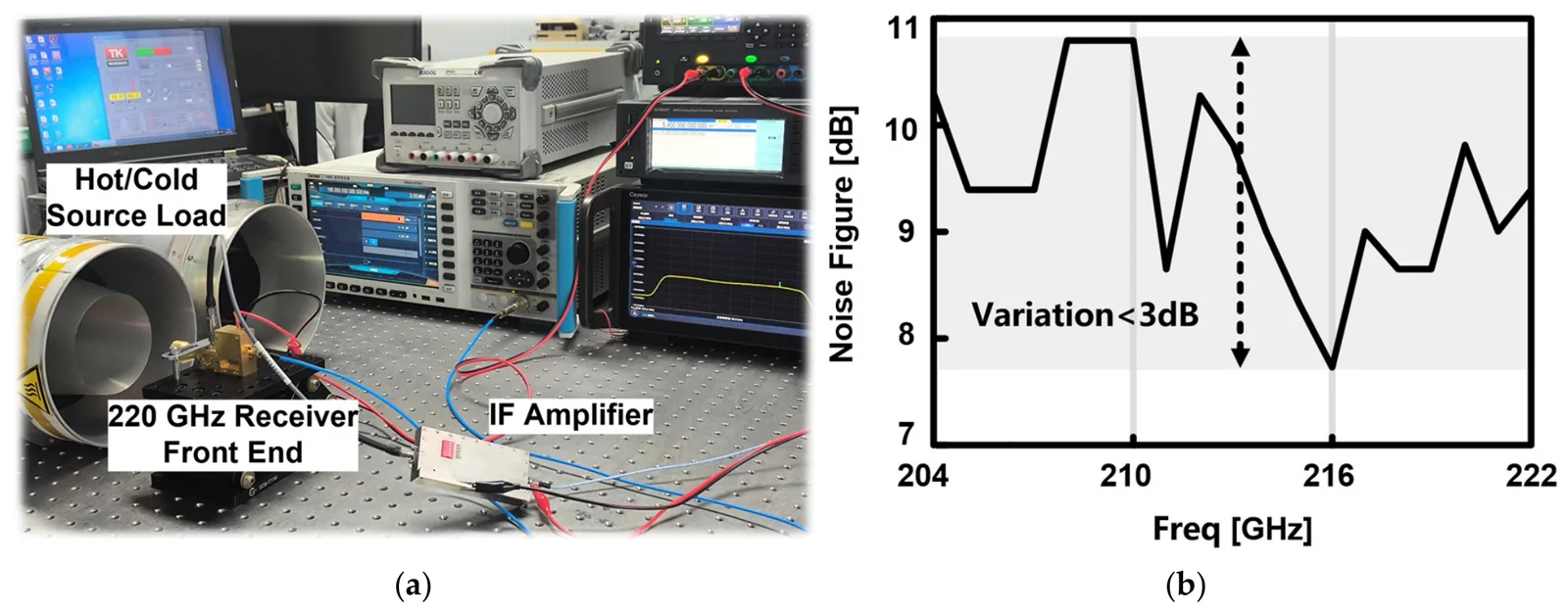
The 220 GHz single-polarization receive front end. (**a**) Measurement setup. (**b**) Noise figure.

**Figure 17:**
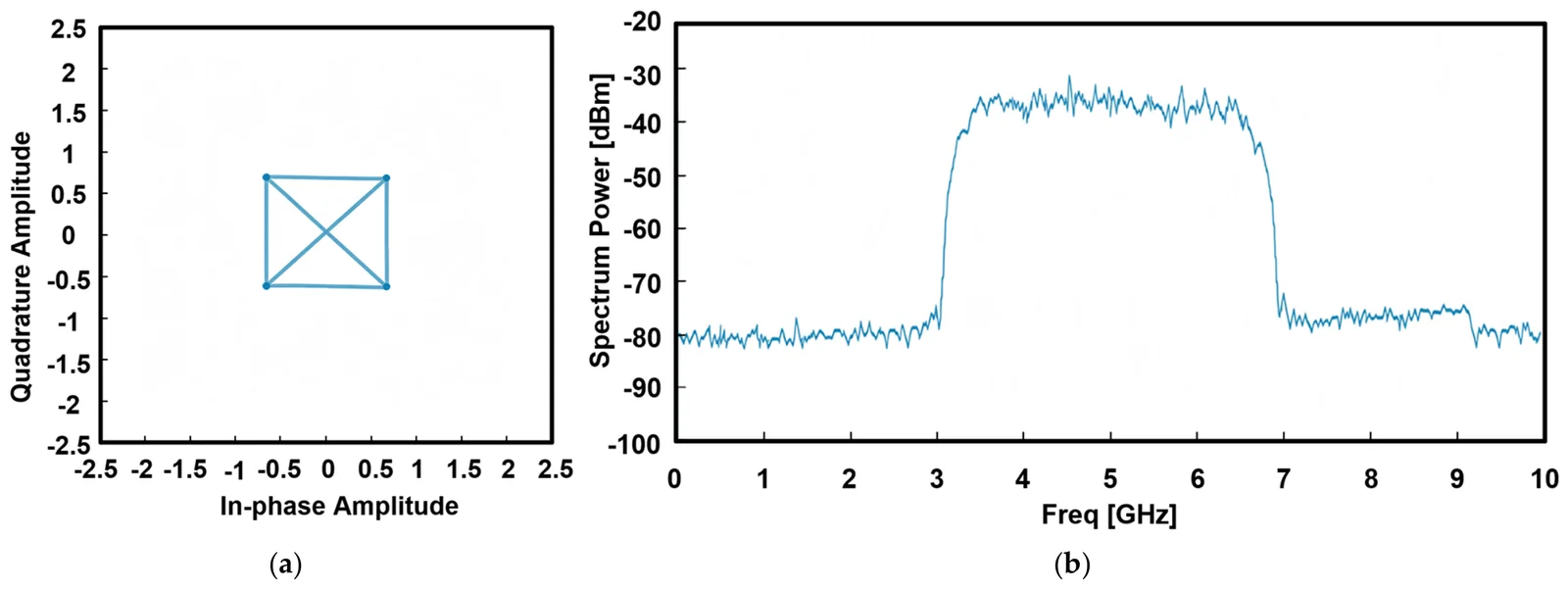
QPSK communication baseband loopback test results: (**a**) Constellation diagram. (**b**) Signal spectrum.

**Figure 18:**
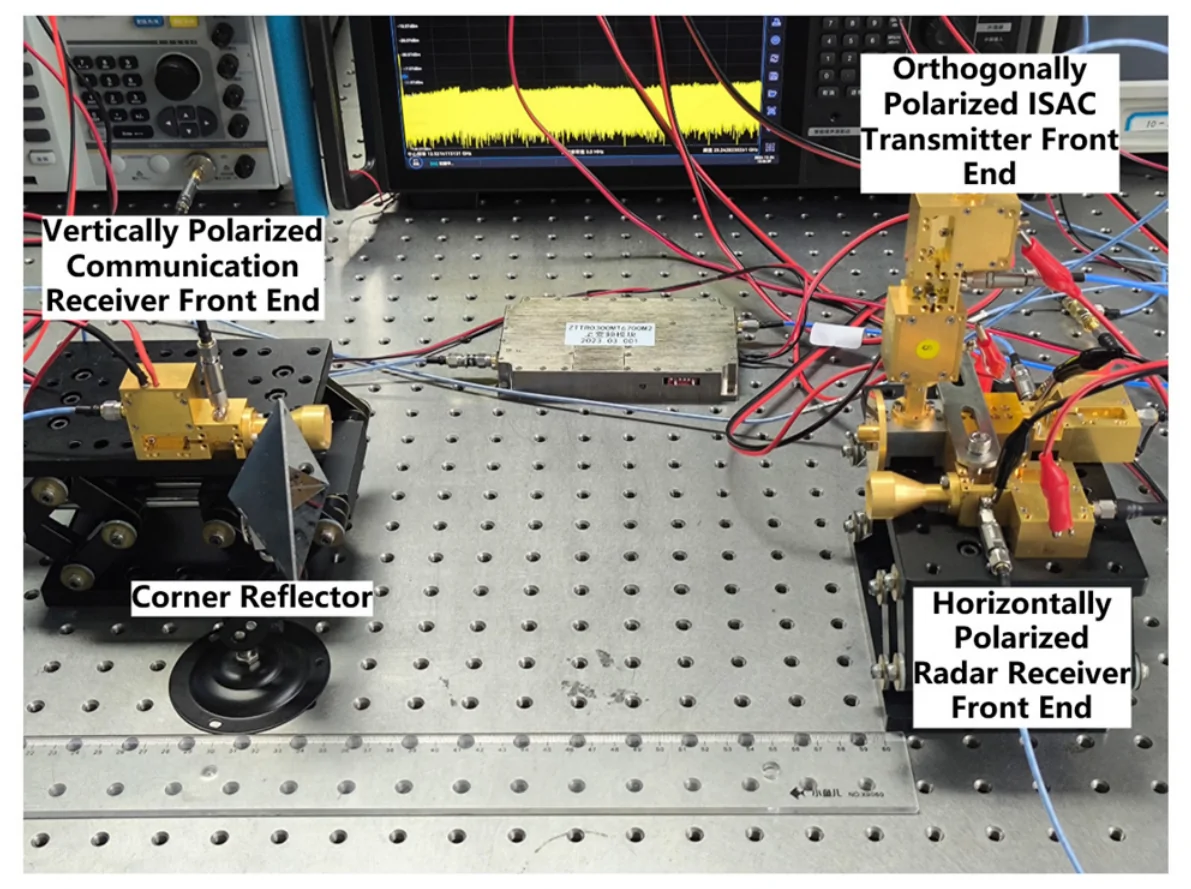
Experimental setup of the 220 GHz orthogonal-polarization-multiplexed ISAC wireless link.

**Figure 19:**
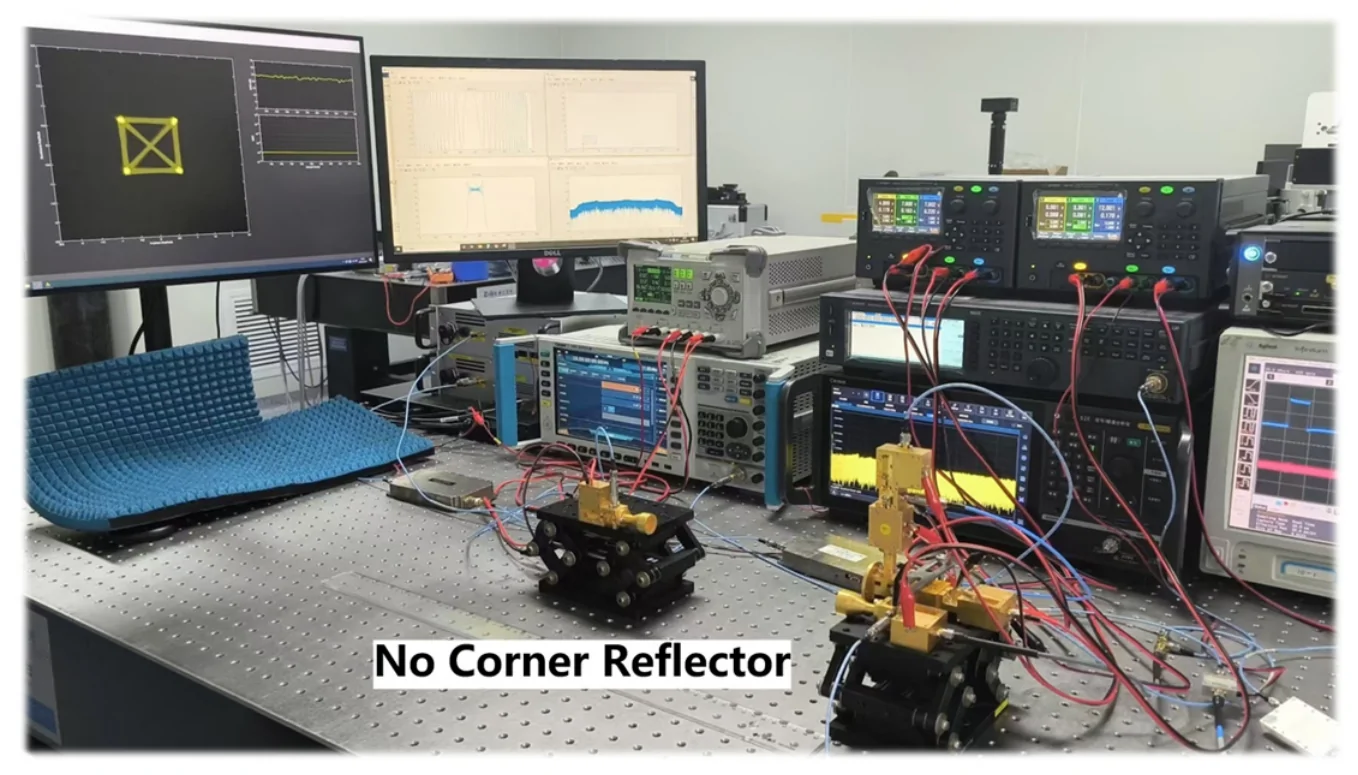
Experimental setup with the radar path terminated by absorbing material and without a pyramidal corner reflector.

**Figure 20:**
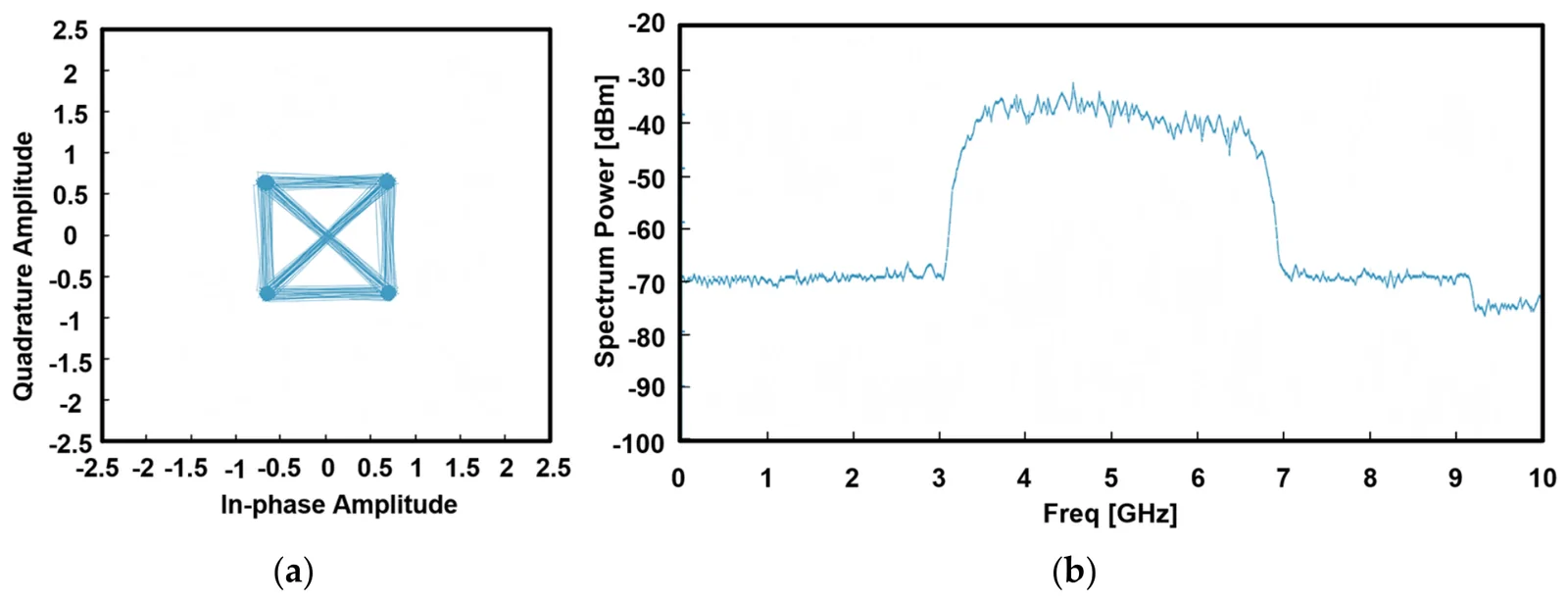
QPSK communication test results without a pyramidal corner reflector: (**a**) Constellation diagram. (**b**) Demodulated signal spectrum.

**Figure 21:**
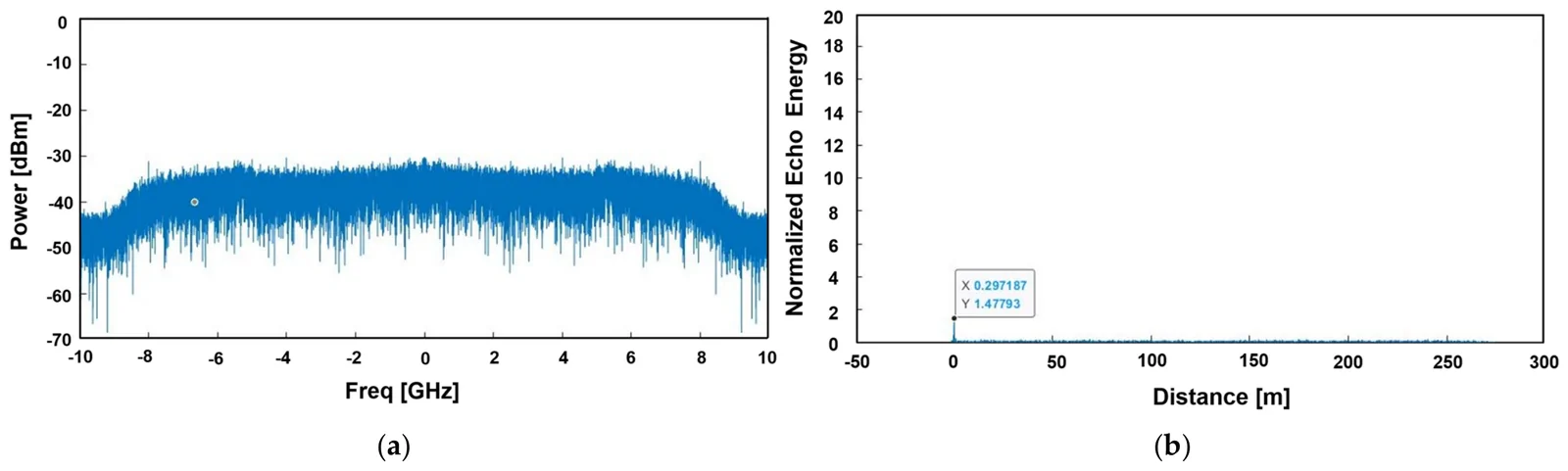
Radar test results without a pyramidal corner reflector: (**a**) Demodulated signal spectrum. (**b**) Ranging profile.

**Figure 22:**
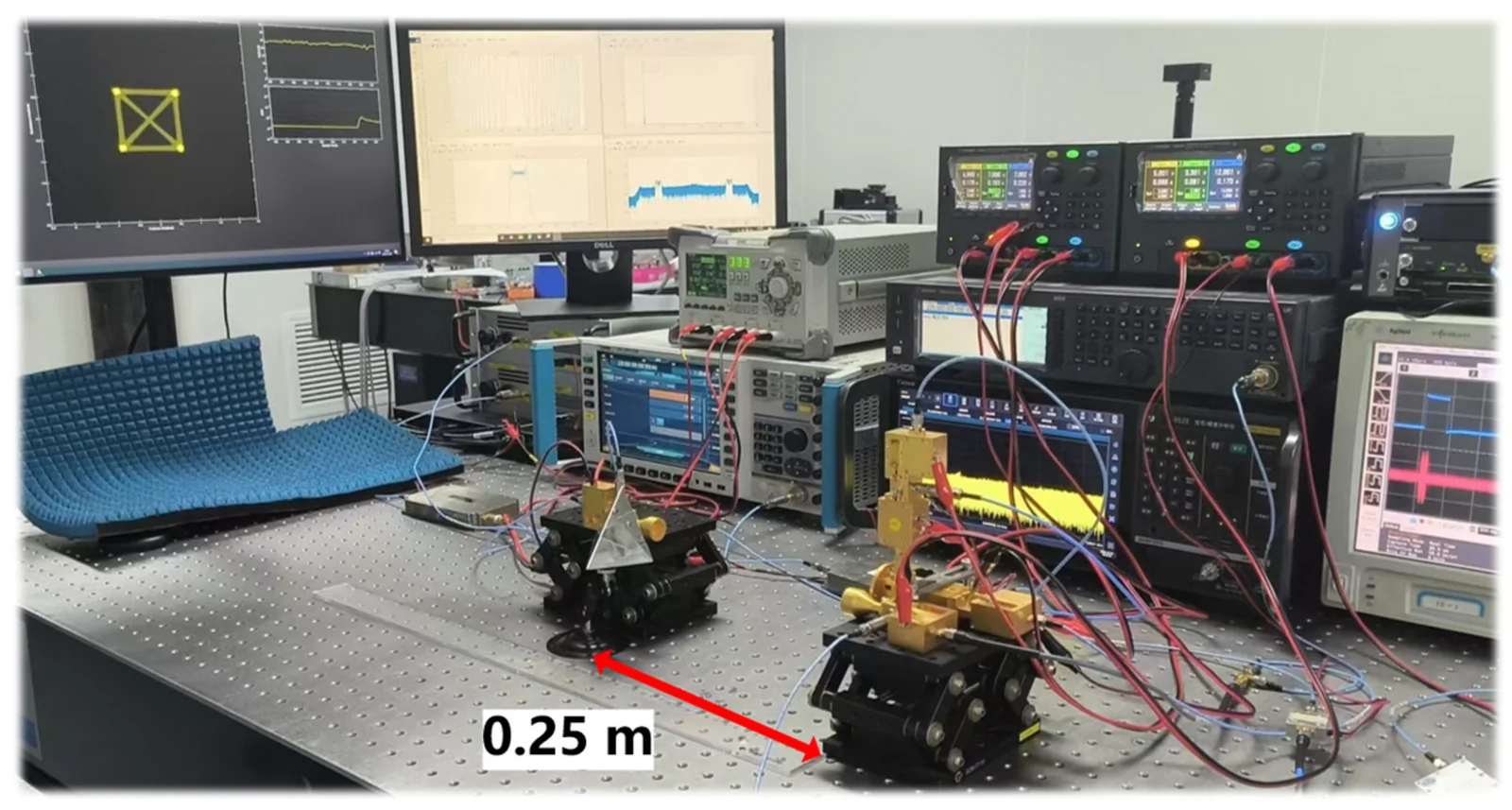
Experimental setup with the pyramidal corner reflector positioned 0.25 m from the radar receiver.

**Figure 23:**
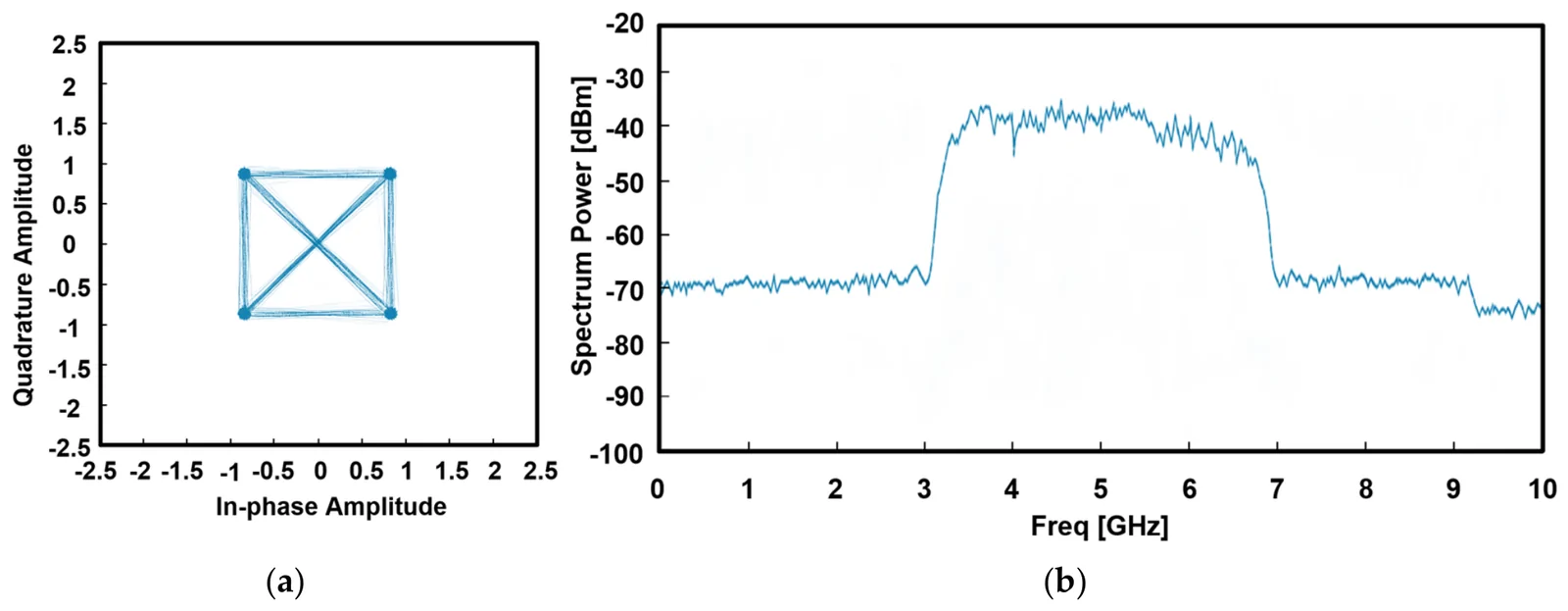
QPSK communication test results with the pyramidal corner reflector positioned 0.25 m from the radar receiver: (**a**) Constellation diagram. (**b**) Demodulated signal spectrum.

**Figure 24:**
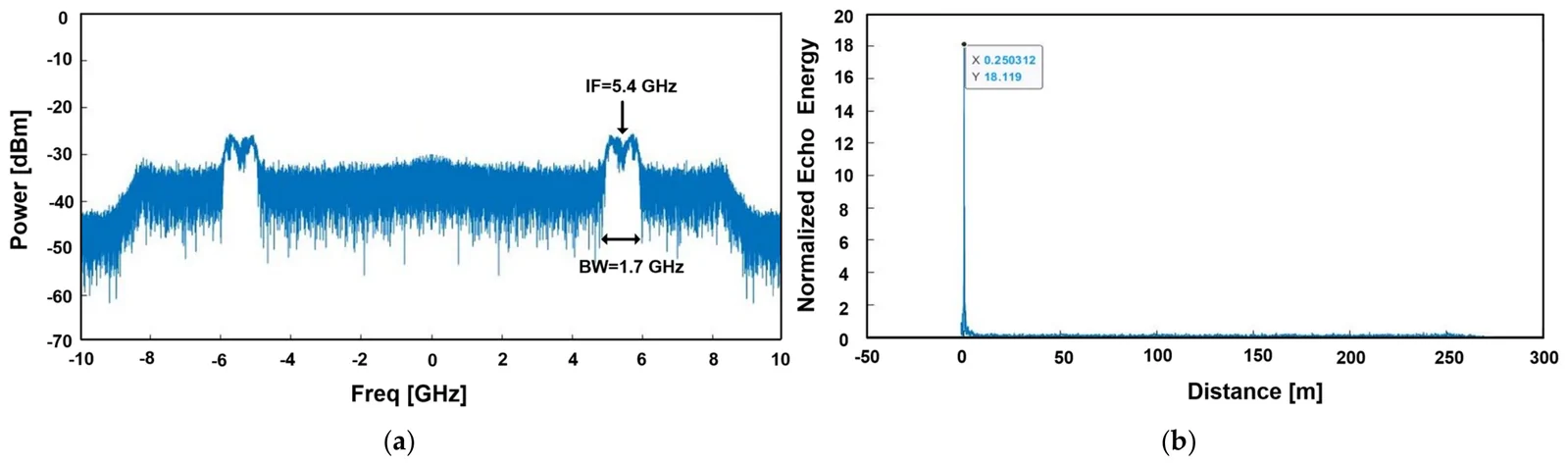
Radar test results with the pyramidal corner reflector positioned 0.25 m from the radar receiver: (**a**) Demodulated signal spectrum. (**b**) Ranging profile.

**Figure 25:**
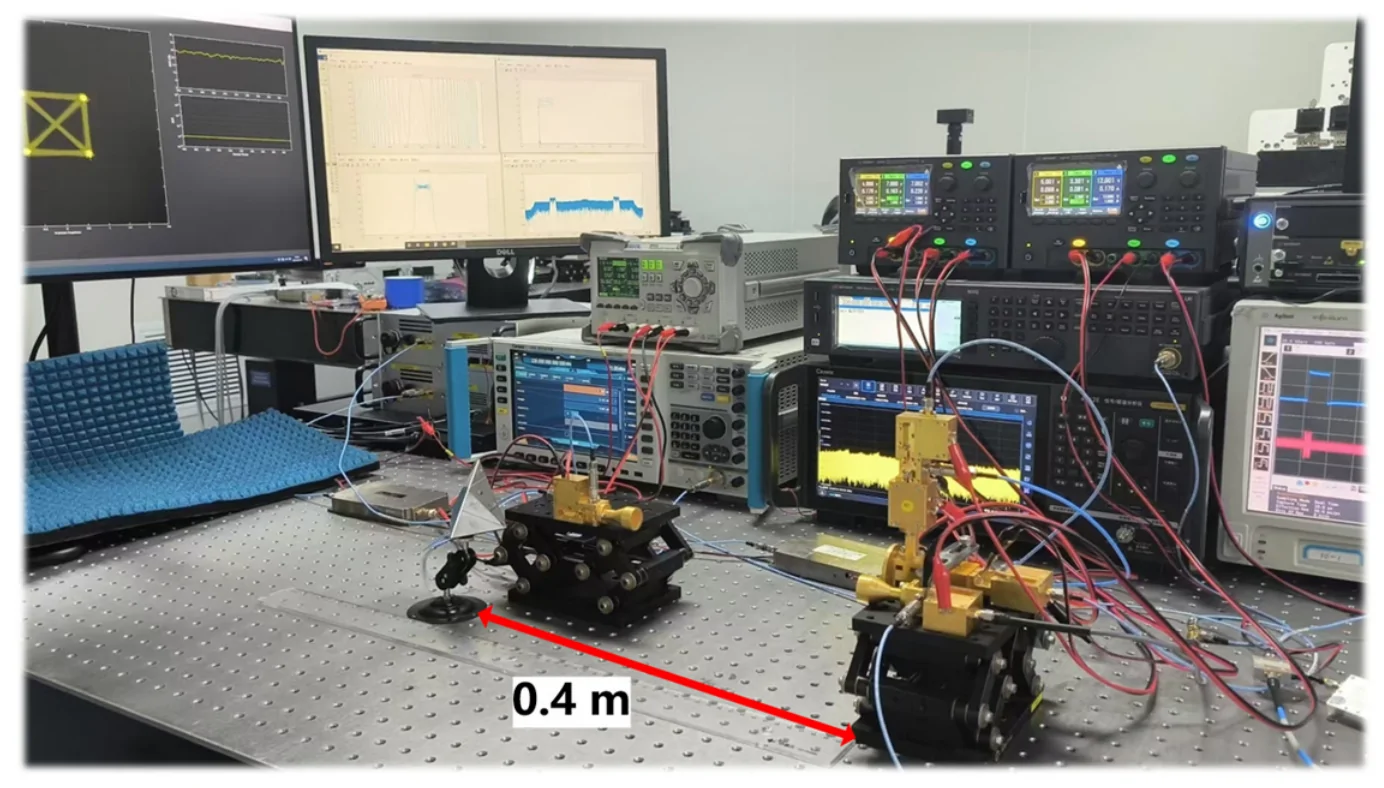
Experimental setup with the pyramidal corner reflector positioned 0.4 m from the radar receiver.

**Figure 26:**
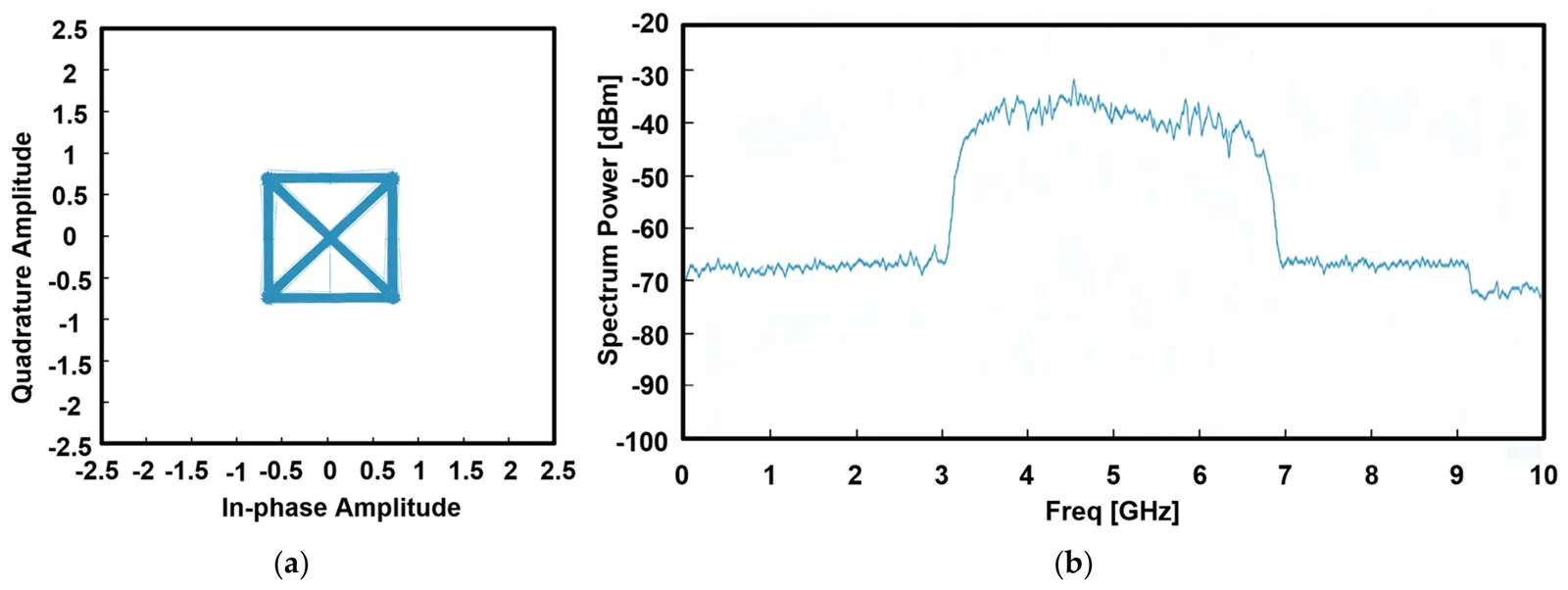
QPSK communication test results with the pyramidal corner reflector positioned 0.4 m from the radar receiver: (**a**) Constellation diagram. (**b**) Demodulated signal spectrum.

**Figure 27:**
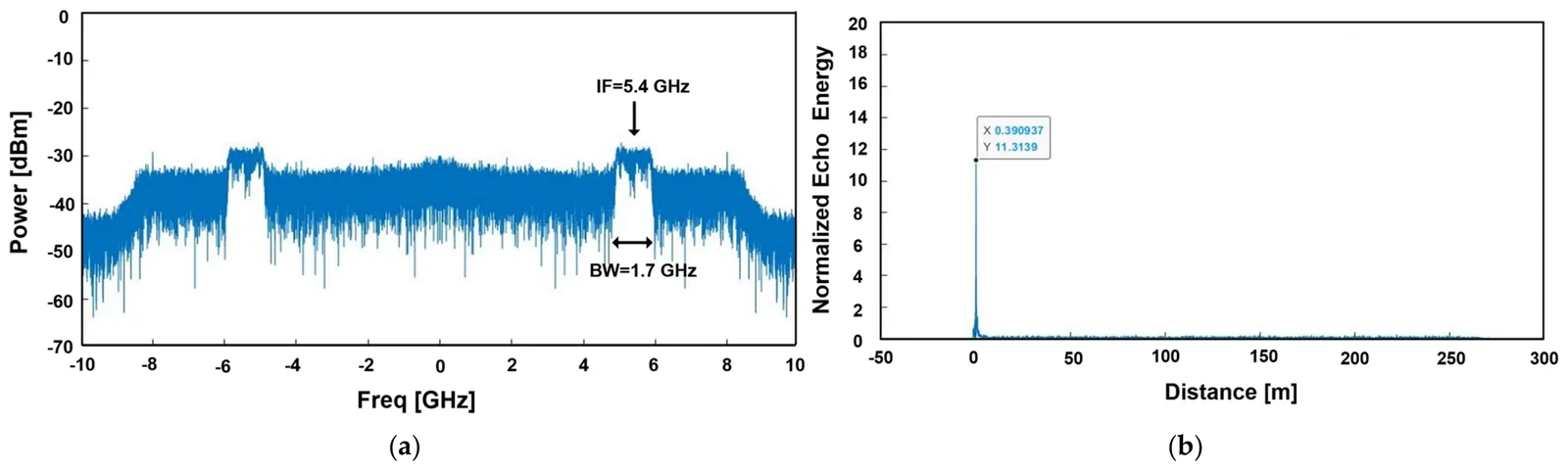
Radar test results with the pyramidal corner reflector positioned 0.4 m from the radar receiver: (**a**) Demodulated signal spectrum. (**b**) Ranging profile.

**Table 1:** Link-budget parameters of the 220 GHz ISAC system.

Parameter	Value
Center frequency	220 GHz
Integrated power of the modulated IF signal	−10 dBm
Free-space path loss over 0.25 m	67.3 dB
Atmospheric attenuation	1 dB
Transmit-chain gain	15 dB
Transmit-antenna gain	14 dBi
Receive-antenna gain	25 dBi
Receive-chain noise figure	9.8 dB
Connection loss	2 dB
SNR requirement for the demodulated communication signal	>24 dB
SNR requirement for the demodulated radar signal	>19 dB

**Table 2:** Performance comparison between the proposed system and related terahertz wireless communication systems.

Reference	Operating Frequency	System Architecture	Functionality	Communication	Sensing
[23]	300 GHz	Polarization multiplexing	Unidirectional communication	100 Gbit/s	None
[24]	220 GHz	MIMO	Unidirectional communication	240 Gbit/s	None
[25]	218 GHz	Polarization multiplexing	Unidirectional communication	41.6 Gbit/s	None
This work	220 GHz	Polarization multiplexing	Integrated sensing and communication	2 Gbit/s	1 cm

## Data Availability

The original contributions presented in this study are included in the article. Further inquiries can be directed to the corresponding author.
